# FGFR as a Predictive Marker for Targeted Therapy in Gastrointestinal Malignancies: A Systematic Review

**DOI:** 10.1007/s12029-025-01214-y

**Published:** 2025-04-09

**Authors:** Nika Seraji, Irina Berger

**Affiliations:** 1 Faculty of Medicine, University of Southampton, Southampton, UK; 2 Department of Pathology, Klinikum Kassel, Kassel, Germany

**Keywords:** Fibroblast growth factor receptor (FGFR), Targeted therapy, Predictive biomarker, Malignant tumour, Gastrointestinal cancers

## Abstract

**Background:**

Gastrointestinal (GI) cancers constitute approximately 25% of cancers worldwide. The fibroblast growth factor receptor (FGFR) family is a promising target for immunotherapy aiming  to enhance survival rates. FGFR alterations are associated with GI carcinomas. Their predictive value in different malignancies remains a focus area. While FGFR inhibitors have been approved for cholangiocarcinoma (CC) therapy, uncertainties remain regarding other GI cancers.

**Methods:**

A systematic review was conducted using the following databases: CINAHL, Embase, Medline, Cochrane Library, PubMed, and Web of Science. The search terms included “FGFR” and each of the GI malignancies. A total of 18 studies were included in this review.

**Results:**

The efficacy of FGFR-targeted therapy is evident. Strong evidence supports the use of FGFR inhibitors in CC, gastro-oesophageal cancer (GC/OC), and hepatocellular cancer, while there is limited evidence for pancreatic cancer (PC) and colorectal cancer (CRC). Alteration forms like FGFR2 fusion or rearrangement are associated with CC, while FGFR2 amplification and FGFR2b overexpression are associated with GC/OC. The administration of multi-kinase inhibitors without prior genomic testing, makes distinct study outcomes not solely attributable to the FGFR blockade.

**Conclusion:**

FGFRs have a predictive value for GI cancers. Certain FGFR alterations are predictable for specific GI cancers. The most established FGFR-targeted therapy is for CC. It is essential to expand the FGFR research field for PC and CRC. Consistent molecular diagnostics in clinical trials are vital to comprehend the patient population with the highest efficacy.

**Graphical Abstract:**

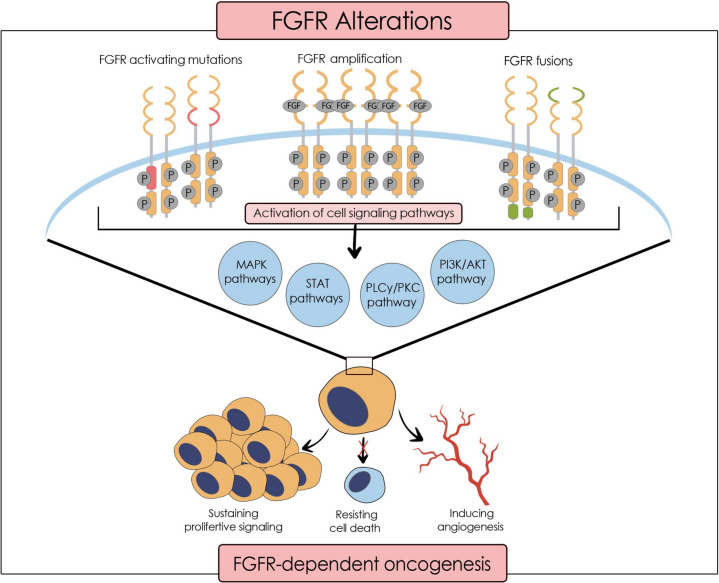

**Supplementary Information:**

The online version contains supplementary material available at 10.1007/s12029-025-01214-y.

## Introduction

Gastrointestinal (GI) cancers are responsible for more than 1 in 4 (26.4%) cancer cases worldwide [1]. The main types of GI cancers are oesophageal, gastric, hepatocellular, bile duct, pancreatic, and colorectal cancer.

The emphasis on early cancer detection stems from the potential higher survival chance by being able to receive possibly a better treatment outcome [2]. Screening programmes, like bowel cancer screening of the NHS [3] and the CDC [4], are an effective approach to identifying early-staged cancers.  National statistics collected by the NHS England Digital [5] state that the 5-year cancer survival in England is lower than 25% for upper GI cancers.

Projections of new cases by Global Cancer Observatory indicate an increase in cancer cases by 2040 especially in countries with a low HDI, mainly due to the ageing population [1, 6]. A more pronounced health and economic burden has been predicted suggesting an urgent need to improve current treatment options and explore potential biomarkers for therapeutic agents [7, 8].

### FGFR—A Potential Marker for Targeted Therapy

FGF is a signalling protein that regulates vital cellular functions in the human body. There are 18 members that bind as a ligand to FGFRs, a family of transmembrane receptor tyrosine kinases, to express their biological function [9, 10]. The four FGFR genes code for the corresponding FGFR 1–4 and the phenomenon of splicing provides multiple isoforms with variable ligand-binding modularity.

**Fig. 1:**
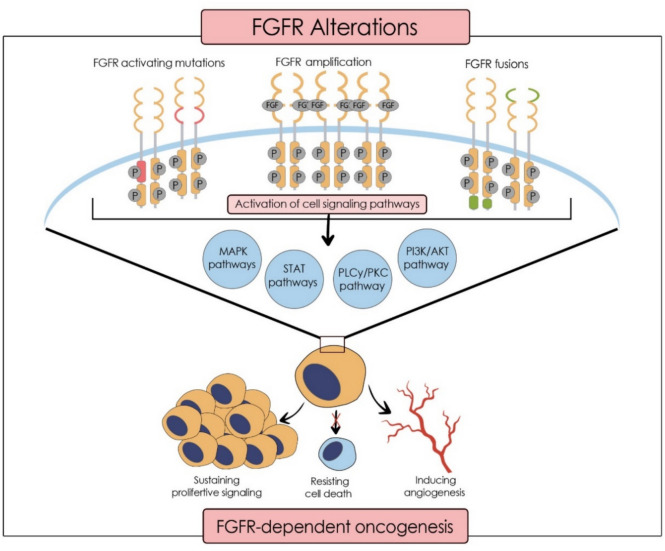
FGFR alterations leading to oncogenesis

The FGF/FGFR signalling involves multiple pathways, including MAPK, P13K/AKT, STAT, and PLC/PKC (Fig. 1). The primary responsibilities of FGFs include tissue repair, regeneration, differentiation, chemotaxis, and proliferation of many cell types such as neurons, smooth muscle cells, adipocytes, chondrocytes, and endothelial cells [10–12]. FGF plays an essential role in angiogenesis and wound healing by producing granulation tissue [10, 11, 13]. Therefore, FGFR inhibition affects both by reducing endothelial adhesion and disrupting tight junctions [14]. Hence, numerous studies have proven that FGFs, mainly FGF1 and FGF2 [12], are highly related to inflammation, by promoting either a pro-inflammatory or an anti-inflammatory response in a vascularization [15] or a chronic disease [16–18]. FGFs act as endocrine hormones and regulate bile acid, fatty acid, glucose, and mineral metabolism [19]. Some members of the FGF family are involved in the embryogenic development of the limbs and neural signalling [12, 20, 21]. However, the research has shown that various FGFs, associated with mitosis of healthy and cancerous cells, orchestrate oncogenesis in different tissues ranging from prostate cancer to hepatocellular carcinoma and colorectal cancer [22–28]. Consequently, FGFR and the signalling pathways must be highly regulated to maintain homeostasis. Alterations or dysregulation in these pathways may lead to carcinogenesis [10, 29, 30].

Conventional chemotherapy and radiation therapy have limitations in efficacy as there is a risk of gaining resistance by the tumour cell or cancer stem cells, respectively [31–35]. Often, patients experience side effects such as nausea, fatigue, alopecia, and threatening neutropenia [36–38]. As a result, the era of precision medicine has emerged as a promising approach to improve the outcomes of cancer patients. Targeted therapy is a branch of personalised medicine targeting agents that maintain the survival of cancerous cells [39]. The hallmarks of cancer, “inducing angiogenesis,” “resisting cell death,” and “sustaining proliferative signalling” provoke uncontrolled cell growth [40] and are promoted by the FGF/FGFR overactivity. The tumour cell needs neovascularization for survival and increased FGFR signalling provides the foundation for vascular formation [14]. FGFRs have been identified as novel therapeutic candidates for cancer therapy [41] since biomarker testing indicated FGFR aberrations in various carcinomas. A recent study on Chinese cancer patients investigated the prevalence of FGFR alterations in 10,582 tumour samples [42]. Previously, an American trial had examined 4853 different neoplasms to detect any FGFR alterations [43]. There has been a similar outcome concerning the prevalence, namely 7% and 7.1% of tumours had FGFR alterations, respectively. Both significantly indicated gene amplifications, particularly in FGFR1, while gene fusions, generally seen in FGFR3, were the least common.

FGFR mutations, also called single nucleotide variants, are changes in the DNA sequence of the FGFR gene. Such variants have been detected in the extracellular, transmembrane, or intracellular domain resulting in an increased affinity for FGF, more frequent dimerization, and more potent kinase activity respectively. The range of single nucleotide variants is remarkably great in various cancer types; therefore, the outcome is highly diverse [44, 45].

An increased number of FGFR gene copies is referred to as FGFR amplification, which leads to an elevated protein expression. This can give rise to “oncogene addiction” described by Bernard Weinstein [46], namely the tumour cells being dependent on the oncogenic protein produced by the pathway for survival [47].

FGFR fusions stem from chromosomal rearrangements of the FGFR gene with other genes during mitosis. This can include gene insertion, deletion, translocation, or inversion, contributing to a fusion protein that activates FGFR without needing a ligand in the daughter cells [48]. Gene fusions have subtype I, causing haematological cancers, and II, resulting in solid tumours. Commonly, type II involves FGFR2-AFF3 and FGFR2-CASP7 gene fusions abnormally activating FGFR2 in breast cancer while type I concerns CNTRL–FGFR1 and ETV6–FGFR3 gene fusions leading to acute myeloid and lymphoid leukaemia patients [41].

The remaining question is: which alteration type is associated with which malignancy? Current research demonstrates specific FGFR aberrations can be found in distinct cancer, such as squamous cell lung carcinoma, where FGFR1 amplification is more prevalent [49]. Equally, studies show that FGFR1 amplifications concern 11.7–14% of breast cancers and are linked to a poor prognosis [43, 50, 51]. Comprehensive genomic profiling could determine predictable FGFR alterations in all GI cancers.

### FGFR Inhibitors—Current Therapeutic Agents

FGFR inhibitors refer to the drugs that target FGFRs, either by blocking their activity or by inducing their degradation. Small-molecule or RTK inhibitors are the main agents interfering with the FGFR signalling pathway. Their mechanism of action is to block the phosphorylation of the tyrosine kinase domain and thereby prevent signal transduction [52]. Recent research implies using antagonistic immunoglobins to target the extracellular domain of FGFRs and FGF ligand traps to inhibit the activity of FGF as a ligand [53, 54].

Small-molecule inhibitors are further classified as selective FGFR inhibitors or multi-kinase (non-selective) inhibitors that are not selective towards a specific RTK. For instance, lenvatinib, as a multi-kinase inhibitor, blocks the tyrosine kinase domain of FGFR1-2, VEGFR2-3, and PDGFR-α/β [55].

Extensive pharmaceutical research has provided selective FGFR inhibitors targeting mainly FGFR receptors, and these include erdafitinib, pemigatinib, futibatinib, and infigratinib. The half-maximal inhibitory concentration (IC_50_) of FGFR inhibitors indicates their affinity and potency to target each receptor. The lower the IC_50_ is, the higher the potency, as less substance is needed to inhibit the FGFR receptor (Table 1). Erdafitinib and futibatinib are pan-FGFR inhibitors as they have a similar affinity for FGFR1-4 [56, 57]. However, infigratinib and pemigatinib show higher potency towards FGFR1-3 than FGFR4 [58, 59].

**Table 1:** IC_50_ in nmol/L of FGFR inhibitors

FGFR Inhibitor	FGFR 1	FGFR 2	FGFR 3	FGFR 4
Erdafitinib [56] (JNJ-42756493)	1.2	2.5	3.0	5.7
Pemigatinib [59] (INCB054828)	0.4	0.5	1.0	30.0
Infigratinib [58] (BGJ398)	1.1	1.0	2.0	61.0
Futibatinib [57] (TAS-120)	1.8	1.4	1.6	3.7
Nintedanib [60] (BIBF 1120)	69	37	108	610
Alofanib [61] (RPT835)	-	< 10	-	-
Anlotinib [62] (AL3818)	11.7	-	-	-
Bemarituzumab [63]	-	Monoclonal Antibody (FGFR2b)	-	-
Fisogatinib [64] (BLU-554)	624	1202	2203	5
Lenvatinib [65] (E7080)	61	27	52	43
FGF401 [66] (Roblitinib)	-	-	-	1.1
Surufatinib [67] (Sulfatinib, HMPL-012)	15	-	-	-
Dovitinib [64] (CHIR258, TKI258)	8	-	9	-
Ponatinib [64] (AP24534)	2.2	1.6	18.2	7.7

According to the NICE guidelines, erdafitinib has been suspended for previously treated FGFR-positive advanced solid tumours in people aged 6 and over [68]. Nevertheless, it is now under investigation for the treatment of individuals with metastatic or unresectable FGFR-positive urothelial cancer [69]. Meanwhile, the US FDA approved it in 2019 for the treatment of adult patients with FGFR2 or FGFR3 alterations and locally advanced or metastatic urothelial carcinoma [70].

Currently, some FGFR inhibitors have been approved for the treatment of CC. The US FDA recommends permigatinib for CC with an FGFR2 rearrangement or fusion [71], while NICE approved it in relapsed or refractory advanced cases, while suspending infigratinib for this purpose [72, 73]. Further, the US FDA proposes futibatinib or infigratinib if the neoplasm is unresectable, locally advanced, or metastatic in adults who have been previously treated [58, 74]. On the other hand, NICE has set a pending status on the approval of futibatinib for treating advanced CC with FGFR2 fusion or rearrangement after systemic treatment [75].

Despite the guidelines made by the US FDA and NICE, it is questionable whether FGFR can serve as a biomarker for other GI cancers and which patients will most likely benefit from the targeted therapy.

New biomarkers need to be examined to detect diverse properties of malignant tumours and suggest potential therapeutic targets [7]. Importantly, there is a gap in the FGFR existing literature. FGFR alterations may be essential elements in cancer therapy. This systematic review will analyze the available literature and evaluate the potential predictive value of distinct FGFR alterations in GI malignancies.

## Aims and Objectives

This systematic review aims to investigate:

- The predictive value of FGFR as a marker in GI carcinomas
- The significance of the type of the FGFR alteration in different GI cancers
- The efficacy of FGFR-targeted therapy
- The establishment of FGFR-targeted therapy for GI malignancies

## Methodology

This systematic review is registered under ERGO II—*Ethics and Research Governance Online* with submission ID: 80,637. The Preferred Reporting Items for Systematic Reviews and Meta-Analyses (PRISMA) 2020 guidelines [76] were adhered to when writing this review.

### Search Databases and Strategy

The literature search was conducted using following databases: the Cochrane Library, Embase (Classic), MEDLINE (Ovid), Web of Science, and CINAHL. “Polyglot,” the search accelerator, was used to translate the MEDLINE (Ovid) search strings systematically into other databases. A non-systematic search of Google Scholar and PubMed was also performed (Supplementary materials). The search was limited to English- and German-language studies published between January 2018 and October 2023 using a combination of keywords. The restriction on the time was due to the need of being precise about new discoveries, given that genomics and oncology are highly emerging fields. The search strategy included a combination of free text and subject heading search using Boolean operators. Database-specific wildcards, truncation, and phrase searching techniques supported the search process.

The search included the combination of these keywords: *FGFR, Fibroblast Growth Factor Receptor, Predictive, Prognostic, Prognosis, Marker, Biomarker, Factor, Targeted Therapy, Treatment, Line-Directed Therapy, Therapeutic, Malignant Tumours, Cancer, Tumour, Tumor, Carcinoma, Malignancy, Neoplasm, Gastrointestinal System, Digestive System, Gastric, Hepatic, Liver, Biliary, Cholangio, Pancreatic, Colorectal.*

### Eligibility Criteria and Selection Process

We conducted this systematic review based on the PICOS framework (P – Population; I – Intervention; C – Comparator; O – Outcomes; S – Study Design) for the eligibility criteria (Table 2).

- Inclusion: Studies in English and German language, clinical trials involving humans subjects, malignancies (CC, GC, HCC, PC, and CRC), adults, children, men, women, period: January 2018–October 2023
- Exclusion: Animal experiments, experiments on cell cultures, case reports, abstract only, studies with a sample size < 10 patients

**Table 2:** PICOS criteria

Population	Men, women, adults, children
Intervention	Patients receiving FGFR-targeted therapy for their primary or secondary malignant tumours (CC, GC, HCC, PC, and CRC)
Comparator	Patients without FGFR alterations or patients receiving other therapies (if applicable)
Outcomes	Prognosis and Efficacy (PR, CR, PFS, OS, and ORR)
Study design	Clinical trials and RCTs

The results were exported to referencing manager EndNote 20 and were de-duplicated electronically and manually. Initial title and abstract screening were done by the authors and an independent party reviewed in accordance with the inclusion and exclusion criteria. All potential papers underwent full-text screening for eligibility based on the inclusion and the exclusion criteria. The eligibility of certain publications was extensively discussed, and inconsistencies were cleared.

### Data Extraction and Synthesis

The following data were extracted from each study: first author, year of publication, study population characteristics (gender, number and median age), study design, tumour type. Variables of interest were the arms of study, outcome parameters, and if applicable genomic testing.

### Quality and Risk of Bias Assessment

The Critical Appraisal Skills Programme (CASP) Checklist [77] was used for assessing risk of bias of RCTs. The modified methodological index for non-randomized studies (MINORS) checklist [78] was applied to assess the included non-RCTs. To avoid reporting bias, quality and risk of bias assessments were conducted by both authors after data extraction.

## Results

### Study Selection

The systematic search led to the identification of 493 papers from CINAHL, Cochrane Library, Medline (Ovid), Embase (Classic), and Web of Science. The non-systematic search of the PubMed database resulted in 42 studies, while Google Scholar and citation searching led to two results.

A total of 109 duplicates were eliminated electronically (53 papers) and manually (56 papers).

**Fig. 2:**
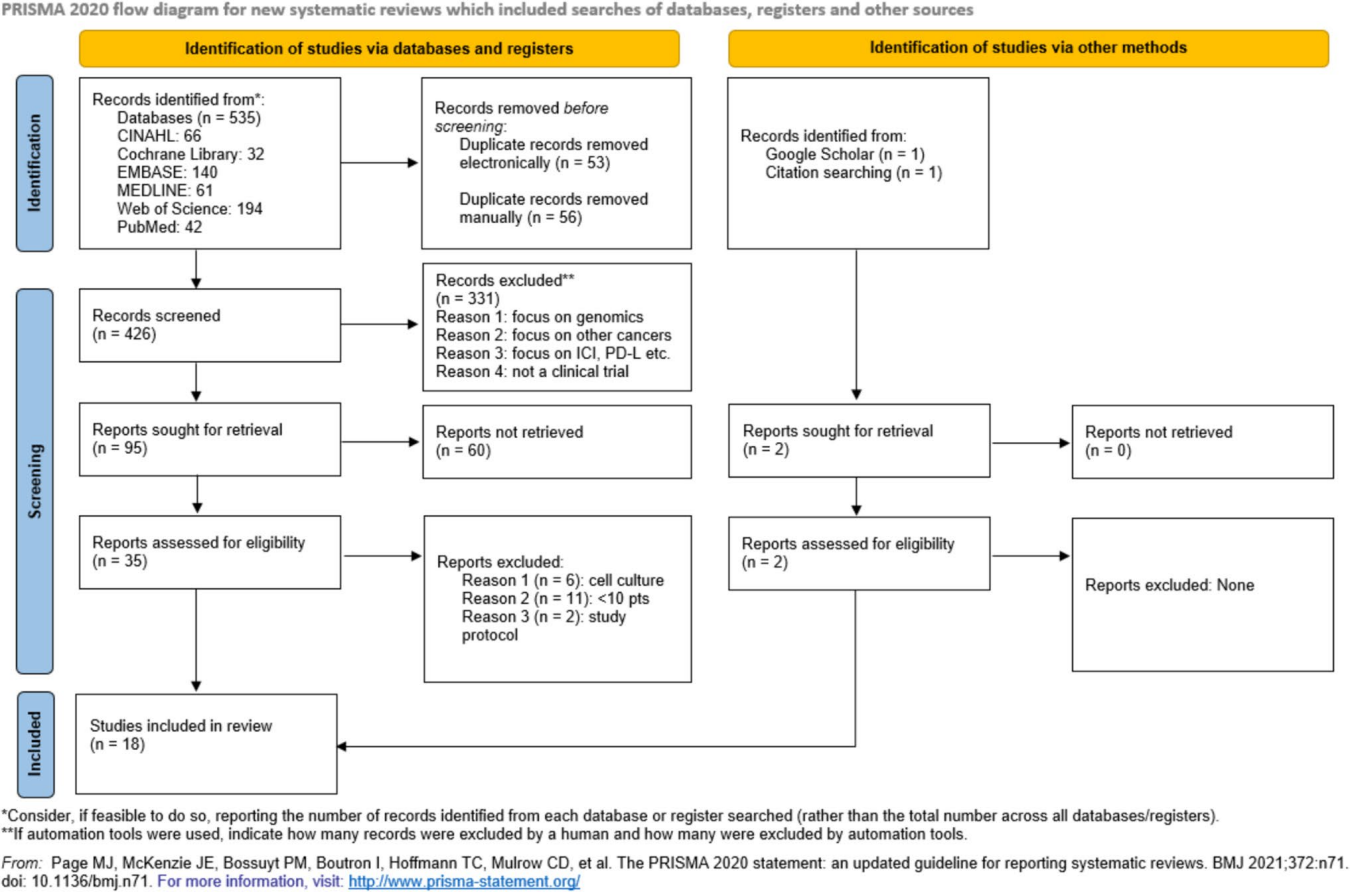
PRISMA flow chart [76] showing the study selection procedure for this systematic review

Screening the title and abstracts of 426 papers according to the inclusion and exclusion criteria led to removing 331 results. The remaining 95 papers were sought for retrieval, only 35 papers were found in full-text. The PRISMA flow diagram [76] illustrates the study selection process (Fig. 2). A total of 18 studies were eligible to be included in this review. It is important to note that no real-world studies were included in the analysis.

### Included Studies to Evaluate FGFR as a Predictive Marker in GI Cancers

The following tables are the study and patients’ characteristics followed by the data extracted from each study (Tables 3 and 4).

**Table 3:** Study and patient characteristics of the clinical trials included in the systematic review

Author/year	Location	Trial design	N pts	M Age	Gender	Tumour
**CC**						
Abou-Alfa et al. [79] 2020	Belgium, France, Germany, Israel, Italy, Japan, Korea, Spain, Taiwan, Thailand, UK, USA	Open-label, single-arm, phase 2	146	59 (26–78)	62 m 84 f	Previously treated, locally advanced or metastatic CC
Javle et al. [80] 2021	USA, Belgium, Spain, Germany, Singapore, Taiwan and Thailand	Open-label, single-arm, phase 2	108	53 (44–64)	41 m 67 f	Unresectable, locally advanced or metastatic CC (with prior chemotherapy)
Goyal et al. [81] 2023	USA, UK, France, Japan, Netherlands, South Korea, Taiwan, Spain, Germany	Open-label, single-arm, phase 2	103	58 (22–79)	45 m 58 f	Unresectable or metastatic FGFR2 fusion or rearrangement positive iCC (with prior systemic therapy)
Shi et al. [82] 2022	China (multiple sites)	Single-arm, phase 2	31	56 (28– 68)	10 m 20 f	Locally advanced or metastatic FGFR2 fusions or rearrangements CC, confirmed (with prior systemic therapy)
Ahn et al. [83] 2022	USA (multiple sites)	Open-label, single-arm, phase 1	12	48.5 (40–66)	3 m 9 f	Advanced CC refractory or intolerant to gemcitabine or fuoropyrimidine chemotherapy
**GC**						
Won et al. [84] 2019	USA	Open-label, single-arm, phase 2	32	59 (35–76)	27 m 5 f	Metastatic or recurrent oesophageal or gastro-oesophageal junction adenocarcinoma
Tsimafeyeu et al. [85] 2023	Russian Federation	Open-label, Phase 1b	21	56 (38–75)	15 m 6 f	Gastro-oesophageal adenocarcinoma, refractory to available therapies
Jiang et al. [86] 2022	China (single site)	Open-label, single-arm,	62	62 (26–86)	37 m 25 f	Advanced, relapsed GC/OC adenocarcinoma (only 1 prior systemic therapy)
Catenacci et al. [87] 2020	USA, South Korea and Taiwan	Open-label, non-randomised, phase 1b and 2	56 (target group)	56 (29–77)	31 m 25 f	Recurrent or metastatic GC/OC adenocarcinoma
Wainberg et al. [88] 2022	China, Hungary, Japan, Republic of Korea, Italy, Taiwan, Turkey, France, Germany, Italy, Portugal, Poland, Russia, Romania, Spain, UK, and USA	Randomised, double-blind, placebo-controlled, phase 2	155	60 (51–64) B 59.5(52–68) P	111 m 44 f	Unresectable or metastatic gastric or gastro-oesophageal junction adenocarcinoma
**HCC**						
Kim et al. [27] 2019	USA, South Korea, UK, Spain, France, Italy, China, Taiwan, Singapore, Germany, Switzerland	Open-label, phase 1	115	61 (18–55)	88 m 27 f	Unresectable HCC (no prior treatment)
Kudo et al. [89] 2018	China, Hong Kong, Japan, South Korea, Malaysia, Philippines, Singapore, Taiwan, Thailand, Belgium, Canada, France, Germany, Israel, Italy, Poland, Russia, Spain, UK, and USA	Randomised open-label, phase 3	954	62 (20–88)	806 m 148 f	Unresectable HCC
Chan et al. [90] 2022	China, France, Germany, Hong Kong, Italy, Japan, Korea, Singapore, Spain, Taiwan, and USA	Phase 1/2	139 HCC	62 (21–85)	118 m 42 f	HCC
Yen et al. [91] 2018	South Korea and Taiwan (multiple sites)	Randomised, open-label, phase 1/2	95	59 (32–84)	83 m 12 f	Advanced HCC not amenable to curative therapy
**CRC**						
Ettrich et al. [92] 2020	Germany (multiple sites)	Randomised, double-blind, placebo-controlled, phase 2	53	63 (37–78)	39 m 14 f	mCRC (1 prior palliative chemotherapy)
Van Cutsem et al. [93] 2018	Argentina, Australia, Austria, Belgium, Canada, Czech Republic, Denmark, France, Germany, UK, Hong Kong, Israel, Japan, South Korea, Luxembourg, Mexico, Poland, Portugal, Russia, Spain, Sweden, Taiwan, Turkey, USA	Randomised, double-blind, placebo-controlled, phase 3	768	62 (22–85)	454 m 314 f	Metastatic or locally advanced colorectal adenocarcinoma not amenable to curative surgery and/or radiotherapy
**PC**						
Xu et al. [94] 2019	China	Single-arm, open-label, phase 1b/2	42 pancreatic	46 (20–70) pancreatic	25 m 17 f	Pancreatic or extrapancreatic NET
Ma et al. [95] 2019	USA	Phase 1b	24 pancreatic	65.5 (42–81)	16 m 13 f	Advanced or metastatic pancreatic or biliary tract adenocarcinoma

**Table 4:** Extracted data from included studies

Author/year	Intervention	ORR (CR/PR)	M OS (months)	M PFS (months)	FGFR status
**CC**					
Abou-Alfa et al. [79] 2020	Pemigatinib	36%—3 CR, 35 PR in FGFR2 fusion and rearrangement cohort	FGFR2 fusion and rearrangement: 21.1 other FGFR alterations: 6.7 No alteration: 4	FGFR2 fusion and rearrangement: 6.9	9% Prevalence of FGFR2 alterations tested by NGS Included in the efficacy: 107 FGFR2 fusions or rearrangements, 20 other FGF/FGFR alterations, 18 no FGF/FGFR alterations. BICC1 was found as the main FGFR2 rearrangement partner (29%) (and in 10 of the ones reaching a response)
Javle et al. [80] 2021	Infigratinib	BICR:23.1%—1 CR, 24 PR I: 30.6–33PR	BICR: 12.2	BICR: 7.3 I: 6.8	FGFR2 status was found by NGS, FISH, or RT-qPCR. 81% had FGFR2 fusions, 19% had other FGFR2 rearrangements. BICC1 was the main FGFR2 fusion partner (25%)
Goyal et al. [81] 2023	Futibatinib	42%—1 CR, 42 PR	21.7	9	324-gene-panel assay: 78% had FGFR2 fusions, 22% had rearrangements. 46 unique FGFR2 fusion partners were identified. Responses occurred in 10 of 24 patients with BICC1 fusions. BICC1 (30%)
Shi et al. [82] 2022	Pemigatinib	IRCC: 50.0%—15 PR I: 40%—12 PR	-	IRRC: 6.3	Molecular epidemiology study: 6.14% prevalence of FGFR2 rearrangements in iCC by FISH. NGS confirmed FGFR2 fusion or rearrangement with Malignant Neoplasms Multi-Gene Analysis Kit. The most common fusion partner was FGFR2-WAC in 3 (10%) patients. BICC1 (6.7%)
Ahn et al. [83] 2022	Ponatinib	9.1%—1 PR (among 11 pts)	15.7	2.4	83.3% of pts had FGFR2 fusion or rearrangements, the rest that FGFR mutations or amplification determined by FISH or NGS
**GC/OC**					
Won et al. [84] 2019	Nintedanib	0%	13.5 (FGFR2)	3.5 (FGFR2)	The MSK-IMPACT NGS assay: 19% of the 27 samples were positive for FGFR2 amplification
Tsimafeyeu et al. [85] 2023	Alofanib	5.26%—1 PR (among 19 pts) at a dose of 50 mg/m^2^	7	3.63	3 of 17 (17.6%) patients had FGFR2 2 + /3 + expression by IHC 2 of 17 (11.8%) patients had FGFR2 amplification by FISH. Only 1 patient was positive by FISH and IHC
Jiang et al. [86] 2022	Anlotinib with toripalimab (PD-1 inhibitor)	32.3%—20 PR (FGFR-M: 40% FGFR-W: 28.6 *p* = 0.37)	All: 11.1 (FGFR-M: 11.1 FGFR-W: 11.1 *p* = 0.31)	All: 4 (FGFR-M: 6.8 FGFR-W: 4 *p* = 0.17)	15 (24.2%) pts had FGFR2-M according to NGS. The FGFR2-M significantly helped target lesion reduction (OR = 0.22; *p* = 0.02). 62.9% of pts had PD-L1 positive cancers
Catenacci et al. [87] 2020	Bemarituzumab	17.9% – 5 PR, high FGFR2b overexpression (*n* = 28) 8.3%—1 PR, low FGFR2b overexpression (*n* = 12)	-	-	28 had tumours with high FGFR2b overexpression, four had moderate expression, 13 had low expression, 11 had no or unknown expression by FISH. (*n* = 52 were evaluable for efficacy)
Wainberg et al 2022	Bemarituzumab + mFOLFOX6 (*n* = 77) vs. placebo + mFOLFOX6 (*n* = 78)	Bemarituzumab: 36/77 (46.8%) Placebo: 26/78 (33.3%) *p* = 0.11	Intervention: - Placebo: 12.9 *p* = 0.027	Intervention: 9.5 Placebo: 7.4 *p* = 0.073	Pre-screening using IHC for FGFR2b overexpression and NGS of cell-free ctDNA for FGFR2 amplification Among 910 pts: 30% were positive for FGFR2b overexpression or FGFR2 amplification. 29% had overexpression of FGFR2b, 38 (4%) had amplification of FGFR2, and 26 (33%) of the FGFR2 amplification cohort had also FGFR2b overexpression 96% of 155 pts had any FGFR2b overexpression, 17% had amplification of FGFR2, and 13% with FGFR2b overexpression also had FGFR2 amplification
**HCC**					
Kim et al. [27] 2019	Fisogatinib	17% in FGF19-positive pts—1 CR, 10 PR (*n* = 66) No response in FGF19-negative or an unknown FGF19 status (*n* = 32) Radiographic tumour reductions in 41% of FGF19 IHC–positive pts	-	FGF19 positive: 3.3 FGF19 negative/unknown: 2.3	Out of 395 samples tested, 27% were positive for FGF19 staining Out of 115, 63% were IHC positive for FGF19. 53 pts with IHC FGF19-positive tumours were assessed for FGFR4 and KLB mRNA expression. 51 pts were positive. FGF19 IHC expression is a marker of FGFR4 pathway activation. 8% of pts were FGF19-positive by FISH
Kudo et al. [89] 2018	Lenvatinib (*n* = 476) vs. sorafenib (*n* = 475)	L: 24.1%—6 CR, 109 PR S: 9.2%—2 CR, 42 PR *p* < 0.0001	L: 13.7 S: 12.3 (HR 0.92, 95% CI 0.79–1.06)	L: 7.3–7.4 S: 3.7 *p* < 0.0001	-
Chan et al. [90] 2022	FGF401 Phase 1: FGF401 single agent (*n* = 61) vs. FGF401 + spartalizumab (*n* = 6 in each arm) Phase 2: group 1 (Asian) (*n* = 30) and 2 (non-Asian) (*n* = 36)	Phase 1: 1 CR, 3 PR (*n* = 59) Arms: (80 mg FGF401 + 300 mg spartalizumab vs. 120 mg FGF401 + 300 mg spartalizumab): 1 PR Phase 2: group 1 (*n* = 28)—2 PR, group 2 (*n* = 31)—2 PR	Phase 1: 5.72 (*n* = 45) – FGF401 single agent	-	Patients had positive FGFR4 and KLB transcript expression by RT-qPCR RT-qPCR and IHC assay tested the FGF19 status. IHC: among patients with HCC given single agent, 27 were FGF19 positive (trend for a better response) and 33 were FGF19 negative 7 FGF19 IHC-positive samples were negative by RT-qPCR and 2 FGF19 IHC-negative samples were positive by RT-qPCR
Yen et al. [91] 2018	Nintedanib (*n* = 63) vs. sorafenib (*n* = 32)	RECIST v.1.1 Nintedanib: 6.3%—2 CR, 2 PR Sorafenib: 3.1%—1 PR mRECIST Nintedanib: 14.3%—2 CR, 7 PR Sorafenib: 18.8%—6 PR	Nintedanib: 10.2 Sorafenib: 10.7 (HR = 0.94, 95% CI 0.59–1.49)	Nintedanib: 2.7 Sorafenib: 3.7 (HR = 1.19, 95% CI 0.73–1.93)	-
**CRC**					
Ettrich et al. [92] 2020	Nintedanib + mFOLFOX6 (*n* = 27) vs. placebo + mFOLFOX6 (*n* = 26)	Nintedanib: 3.7%—1 PR Placebo: 3.8%—1 PR (*p* = 1.0)	Nintedanib: 17.1 Placebo: 9.9 (*p* = 0.9387)	Nintedanib: 8.1 Placebo: 4.6 (*p* = 0.22)	-
Van Cutsem et al. [93] 2018	Nintedanib (*n* = 386) vs. placebo (*n* = 382)	0%	Central review: Nintedanib: 6.4 Placebo: 6 (*p* = 0.8659) I review: by baseline n of metastatic sites and rectum as site of primary tumour (*p* > 0.05)	Central review: Nintedanib: 1.5 Placebo: 1.4 (*p* < 0.0001) I review: Nintedanib: 2.6 Placebo: 1.4 (*p* < 0.0001)	Exploratory biomarker analyses are ongoing—no information was given at the time of publishing
**PC**					
Xu et al. [94] 2019	Surufatinib (pancreatic NET)	Tumour shrinkage from baseline > 10% in 61% of the pancreatic NET pts I:19%—8 PR Independent assessment: 12%—5 PR	-	I: 21.2 Independent assessment: 19.4	ELISA measured plasma concentrations of bFGF, FGF23 + other TKR. Treatment led to an increase in plasma FGF23 levels from baseline. No significant change in bFGF levels. Lower baseline levels of bFGF were significantly associated with longer PFS
Ma et al. [95] 2019	Dovitinib + gemcitabine + capecitabine	3 PC pts had a PR (*n* = unknown)	15 PC pts received treatment as first-line therapy (M OS:13.3) 18 PC pts were naive to gemcitabine. (M OS: 9.5)	-	Sandwich ELISA kit measured total FGF23 in plasma. Quantikine® ELISA kit analysed other biomarkers No relationship was detected between markers’ baseline plasma level and clinical outcome. The plasma FGF23 levels increased in 4 of 5 pts with a PR after 19 days of dovitinib. It is unclear how many of these had PC

### CC

All studies were single-arm phase 2 studies, except for the Ahn et al. study [83], published between 2020 and 2023. Three trials were multinational [79–81]—one study was conducted in China [82] and one in the USA [83]. Participant numbers ranged from 12 to 146 with a similar median age (48.5–59 years), predominantly including women.

Genomic testing has been performed by all studies. Most trials used NGS with different kits, except for one study [81], leading to the conclusion that the FGFR2 fusion and rearrangement is the most prevalent FGFR2 alteration in CC. Other methods including FISH [80, 82, 83] and RT-qPCR [80] were also used for FGFR2 detection. Two papers distinguished between fusion and rearrangements finding that most of the tumours have FGFR2 fusions [80, 81]. Testing for fusion partners showed in many studies that BICC1 is the main fusion partner [79–81]; yet, it did not correlate with the response rate [79]. However, Shi et al. found WAC as the most common fusion partner, namely in 10% of the cohort [82]. In the international trial of Goyal et al., this was the second most common fusion partner along with KIAA1217 in only 3.75% of the patients [81]. Abou-Alfa et al. conducted a molecular epidemiology study—9% of CC have FGFR2 alterations [79]. More specifically, Shi et al. tested 717 iCC tumour samples, 6.14% had detectable FGFR2 rearrangements by FISH [82].

The medications administered were pemigatinib, futibatinib, infigratinib, and ponatinib. The FIGHT-202 study investigated pemigatinib’s impact on patients with FGFR2 fusions or rearrangements, with other FGFR alterations and compared the outcomes with patients without FGFR alterations. The only responders were FGFR2 fusions and rearrangements patients with an ORR of 36%, three had CR and 35 had PR. This was the trial with the highest complete responders [79]. Shi et al. also administered pemigatinib to a smaller sample size (30 patients) in China. Comparing these two trials, Shi et al. had a higher investigator- (40%) and IRCC-assessed (50%) ORR [82]. Although the investigator-assessed median PFS was not reached, the IRRC-assessed median PFS was 6.3 months [82], being comparable to the FGFR2 fusion and rearrangement cohort in FIGHT-202 (6.9 months) [79]. The median OS in the FIGHT-202 study was majorly higher for patients with FGFR2 fusion and rearrangement (21.1 months) than other alterations (6.7 months) or no alterations (4 months) [79].

Javle et al. analyzed the FGFR2 status in 96 patients through NGS, FISH, or RT-qPCR. Eighty-one percent had FGFR2 fusions and 19% had other FGFR2 rearrangements. Administering infigratinib led to a comparable BICR-assessed and investigator-assessed median PFS (7.4 vs. 7.3 months) and median OS (11.8 months vs. 12.2 months). BICR observed 23.1% ORR, 1 CR and 24 PR, while the investigator-assessed ORR was 30.6% with only PR [80].

Goyal et al. performed genetic testing with a 324-gene-panel assay, discovering that 78% had FGFR2 fusions, and the rest had rearrangements. In those with FGFR2 fusions, fusion partners were found, 30% had BICC1 as fusion partner. Ten of 24 patients (42%) with BICC1 fusions and 25 of 56 patients (45%) with non-BICC1 fusions had a response. The ORR with futibatinib was 42%, including 1 CR and 42 PR. The median PFS was 9 months, and the median OS was 21.7 months, the highest among the reviewed CC studies [81].

Ahn et al. had the smallest cohort and intervened with ponatinib. Ten out of 12 patients had FGFR2 fusion or rearrangement tested by FISH or NGS. The trial resulted in the lowest ORR (9.1%) and lowest median PFS (2.4 months). The median OS was 15.7 months [83].

### GC/OC

Studies published between 2019 and 2023 explored gastric adenocarcinoma [85], gastro-oesophageal junction adenocarcinoma [87, 88], or both [86, 88]. Some trials were multinational [87, 88] and others in a single country [84–86]. Population size ranged from 21 to 155 patients and the median age spanned from 56 to 62 years.

Each study administered a different FGFR inhibitor except for Catenacci et al. and Wainberg et al. who administered bemarituzumab [87, 88]. Four papers concerned FGFR2 amplification and FGFR2b overexpression [84, 85, 87, 88], while Jiang et al. focused on FGFR2 mutation [86]. Detection methods used were NGS [84, 86, 88], IHC [85, 88], and FISH [85, 87]. In one case, there were inconsistencies between the testing method [85].

All studies performed genetic testing. Wainberg et al. analyzed 910 patients prior to enrolment. Thirty percent of those tested positive: 29% had FGFR2b overexpression, 4% had FGFR2 amplification, and 33% of those with amplification also showed FGFR2b overexpression. This study randomised 155 patients, 96% had FGFR2b overexpression, 17% had FGFR2 amplification, and 13% of the patients in the FGFR2b overexpression cohort had both FGFR2b overexpression and FGFR2 amplification [88].

Across all reviewed GC/OC studies, Won et al. achieved the shortest median PFS (3.5 months) along with reaching no response by administering the multi-kinase inhibitor, nintedanib. Nineteen percent of their 27 samples had FGFR2 amplification. Patients with FGFR2 amplifications tended to have a longer PFS than patients without such alterations (3.5 vs. 1.9 months, p = 0.92), but the difference was statistically insignificant. However, the three longest PFS (> 8 months) were in tumours without FGFR amplification. This study had the longest median OS among GC/OC studies being 13.5 months for the patients with FGFR2 amplification [84].

Tsimafeyeu et al. tested 17 tumour samples and found only one patient positive for FGFR2 amplification by both FISH and IHC. A percentage of 17.6 were positive by IHC and 11.8% were positive FISH. The study administered alofanib at different doses and reached an ORR of 5.26% at 50 mg/m^2^ among 19 evaluable patients (1 PR). The median OS was 7 months and the median PFS was 3.63 months like Jiang et al. (4 months) [85].

Combining anlotinib and a PD-1 inhibitor was done by Jiang et al., and they investigated regarding FGFR2 mutations. A percentage of 24.2 had FGFR2 mutations, and these tumours were significantly associated with target lesion reduction (OR = 14, *p* = 0.02). The ORR was 32.3% with 20 PR among all patients enrolled. Although it was statistically insignificant, there was a trend of FGFR2-M having a higher PFS and ORR than FGFR2-W after anlotinib [86]. The median OS was 11.1 months in the main, FGFR2-M and FGFR-W cohorts being the highest among the reviewed trials within this group [86].

Considering bemarituzumab, one multinational trial co-administered it with chemotherapy comparing it to sole chemotherapy agent mFOLFOX6. FGFR2b monoclonal antibody showed higher ORR (46.8% vs. 33.3%) and median PFS (9.5 months vs. 7.4 months); yet, these were statistically insignificant. They did not achieve a median OS in the intervention group; yet, in the placebo group, it was 12.9 months (*p* = 0.027) [88].

Catenacci et al. used FISH to differentiate the gradients of FGFR2b expression. Twenty-eight patients had high FGFR2b overexpression, four had moderate expression, 13 had low expression, and 11 had no or unknown expression. The ORR was 17.9% (5 PR) in high and 8.3% (1 PR) in low FGFR2b overexpression group. No response was seen in the moderate and no/unknown FGFR2b overexpression cohort [87].

### HCC

The four studies on HCC were published between 2018 and 2022. Three studies were conducted internationally [27, 89, 90], one was in South Korea and Taiwan [91]. Participant numbers ranged from 95 to 954, with mainly male participants. Their median ages were comparable (59–62 years).

The two studies administering a predominantly FGFR4 inhibitor, performed genomic testing [27, 90]. Kim et al. utilized IHC and FISH to determine FGF19 positivity of the tumours retrospectively, followed by testing those positive tumours on FGFR4 and KLB expression. Out of 395 samples tested by IHC, 27% were positive for FGF19 staining (≥ 1%). They had 115 patients and 63% were IHC-positive for FGF19. Ninety-six percent of 53 IHC FGF19-positive tumours were assessed for FGFR4 and KLB mRNA expression. Ninety-six percent were positive [27]. Chan et al. conducted a dose escalation and dose expansion trial [90]. They only recruited patients positive for FGFR4 and KLB transcript expression by RT-qPCR. The FGF19 status was additionally tested by IHC. A trend for better response had been observed among the IHC FGF19-positive patients treated solely by FGF401. Again, here were inconsistencies between the testing methods. Seven IHC FGF19-positive samples were negative by RT-qPCR, and two FGF19 IHC-negative samples were positive by RT-qPCR. No statistically significant association was identified between FGF19-positive tumours and poor prognosis (*p* = 0.13) [90]. Fisogatinib resulted in 17% ORR (1 CR and 10 PR) in 66 FGF19-positive tumours. The patient experiencing a CR was in the 600-mg cohort. One patient experienced a PR in the 280-mg cohort, two in the 420-mg cohort, and seven in the 600-mg cohort. No response was seen in FGF19-negative patients or with an unknown FGF19 status confirming the FGFR4 pathway integration. Forty-one percent of IHC FGF19-positive patients had a radiographic tumour size reduction. The median PFS was longer for IHC FGF19-positive tumours (3.3 months) compared to IHC FGF19-negative tumours (2.3 months) [27]. In phase 1, a single agent FGF401 achieved an ORR of 6.8% (1 CR and 3 PR) among 59 HCC patients. The median OS was 5.72 months assessed in 45 patients. There was 1 PR in each of the combination arms of FGF401 + spartalizumab making an overall ORR of 8.33% [90]. During phase 2, in both Asian and non-Asian groups, two patients achieved a PR leading to the same ORR as the single agent in phase 1 [90].

No genetic testing was performed to determine the FGFR status in the RCTs [89, 91]. Kudo et al. randomized patients into a lenvatinib or sorafenib arm via a concealment method in a 1:1 ratio [89]. The masked independent review calculated the median PFS as 7.3–7.4 months in the lenvatinib arm, double that of the sorafenib arm (*p* < 0.0001). The ORR was statistically significantly (*p* < 0.0001) higher with lenvatinib (24.1% vs. 9.2%). The results for median OS were statistically insignificant [89]. Yen et al. performed a 2:1 randomization into a nintedanib and sorafenib arm [91]. The median OS was 10.2 months for nintedanib, which was comparable to sorafenib (10.7 months). The median PFS was 2.7 months for the nintedanib group, being lower than the sorafenib arm by a month. The ORR according to RECIST v.1.1 was calculated for nintedanib around 6.3% (2 CR and 2 PR) which was higher compared to sorafenib being 3.1% (1 PR). The mRECIST results were different as nintedanib had a lower ORR with 14.3% (2 CR, 7 PR), while sorafenib had 18.8% (6 PR) [91].

### CRC

Both publications on CRC were RCTs [92, 93] published between 2018 and 2020. They were conducted in Germany [92] or internationally [93]. The number of participants varied from 53 to 768, predominantly male, with a median age of 62 to 63 years. None of the studies included results of FGFR status testing.

Ettrich et al. and Van Cutsem et al. investigated nintedanib’s effects randomizing patients into FOLFOX + nintedanib vs. FOLFOX + placebo [92] and nintedanib vs. placebo [93], respectively. Although the results were statistically insignificant, the median OS (difference of 7.2 months) and PFS (difference of 3.5 months) were numerically higher in the nintedanib + FOLFOX group. Unlike Van Cutsem et al., who did not have any responders [93], the ORR in Ettrich et al. study was 3.7% and 3.8% in the intervention vs. placebo cohort, respectively, with each 1 PR, yet statistically insignificant (*p* = 1.0) [92].

Van Cutsem et al. reported a statistically significant longer PFS in the intervention group according to the central review by 0.1 months (*p* < 0.0001) and to the investigator review by 1.2 months (*p* < 0.0001) [93]. The OS seemed numerically higher with nintedanib than with placebo; yet, it was statistically insignificant [93]. No association was identified between OS and either the baseline number of metastatic sites (1 vs. > 1) or the rectum as the primary tumour site. Nintedanib favours in cases with more than one metastatic site; yet, it cannot be ruled out that there is no difference at 95% Cl [93].

### PC

Only two PC studies matched the inclusion and exclusion criteria. Both phase 1b studies were published in 2019. A Chinese neuroendocrine study administered surufatinib to 42 pancreatic NET patients [94]. Another trial co-administered dovitinib with gemcitabine and capecitabine to 24 pancreatic cancer patients in the USA [95]. The median age was 46 and 65.5 respectively. Both studies applied the ELISA kit to measure bFGF and FGF23 levels at baseline and during treatment. These trials enrolled predominantly men.

The pancreatic NET cohort had investigators’ and independent assessments [94]. They were variable regarding ORR of 19% vs. 12% (8 PR vs. 5 PR, respectively) [94]. A > 10% tumour shrinkage from baseline was observed in 61% of patients in the pancreatic NET cohort [94]. The investigator calculated a PFS of 21.2 months, while independent reviewers assessed it to be 19.4 months [94].

Ma et al. reported that the pancreatic cancer patients (*n* = 15) receiving dovitinib as first-line therapy, their median OS was 13.3 months, compared to the patients (*n* = 18) enrolled naïve to gemcitabine who had a shorter median OS, namely 9.5 months [95].

The plasma FGF23 levels increased in both studies. It increased significantly from baseline in best response patients with surufatinib (*p* < 0.0001) [94]. Dovitinib intervention resulted in four out of five patients having an increased FGF23 level from baseline during the first cycle of treatment [95]. However, both trials did not define how many of the patients had PC. Xu et al. examined 17 patients with pancreatic and 19 patients with extrapancreatic NETs finding no significant change in bFGF levels in best response or progressive diseases patients. However, lower baseline levels of bFGF were significantly associated with prolonged PFS rising from 16.7 to 21.15 months [94].

### Quality Assessment

The methodological quality assessment and risk of bias were divided into two groups, using CASP checklists for RCTs [77] and the modified MINORS criteria for non-RCTs [78]. Some papers disclosed receiving funding from pharmaceutical companies [80, 81, 95], biotechnology companies [88], and medical clinics [83].

Four RCTs were reviewed according to the CASP checklist for RCT [77] quality assessment (Supplementary materials). Whether the benefit outweighs the harm and cost is difficult to assess as the cost of this treatment is unknown; yet, targeted therapy is known to be a high-cost therapy. The benefit is defined as efficacy. Not blinding the assessors or participants may lead to performance bias. The modified MINORS criteria [78] (Supplementary materials) showed that the 13 non-RCTs, especially those concerning CC [79–82], were of good quality. The lowest score was 14/18 [84]. The main potential performance biases were the uncertainty or the absence of the assessor’s blindness and no prospective calculation of the sample size. Thus, the objectivity of the results may be compromised.

## Discussion

The significance of FGFR has been evaluated across various GI cancers with trials demonstrating notable efficacy of FGFR-targeted therapy [79–82, 85, 87]. Nevertheless, the depth and the quality of the available clinical trials differs depending on the cancer type.

Distinct FGFR alterations were identified as predictive biomarkers for the therapy of certain GI cancers. All reviewed CC studies in highlighted FGFR2 fusion or rearrangement as the predominant type of alterations in CC [79–83]. As a genomic profiling study of iCC elaborates, the most common FGFR2 fusion partner is BICC1, observed in 40.7% of the samples [96]. This finding aligns with the reviewed CC studies that reported it in 25–30% of the cases [79–81]. Demographic differences may contribute to finding different fusion partners as in the Shi et al. trial [82]. FGFR-targeted therapy is best established for CC since FDA and NICE guidelines recommend FGFR inhibitors for the treatment of CC [58, 71–74]. In GC/OC, FGFR2 amplification and FGFR2b overexpression may be the main alterations [84, 85, 87]. A single study explored FGFR2-M [86] providing a different perspective. The importance of genomic testing was elicited in trials administering multi-kinase inhibitors such as anlotinib and nintedanib [84, 86]. Molecular testing demonstrated FGFR’s predictive value through observed efficacy in patients with FGFR alterations [84–88]. Phase 2 and 3 trials are needed to confirm the findings and strengthen the evidence for GC/OC. Aberrant FGF19-FGFR4 signalling plays a significant role in hepatocellular oncogenesis [27, 90]. FGF19-positive patients showed radiographic tumour shrinkage and a trend for better responses to FGFR4 inhibitors [27]. Dose-dependent responses observed with fisogatinib, demonstrated FGF19-positive tumours as the responsive population [27]. These findings establish FGFR4 as a promising therapeutic target in FGF19-positive HCC. The evidence presented by the studies, establishes a strong predictive value of FGFR alteration in GC/OC, HCC, and CC. Most studies found statistically and clinically significant results, recruited a wider pool of patients, and applied molecular diagnostics [79–82, 85, 87].

However, a conclusive predictable FGFR alteration has not been identified for all GI carcinomas. For instance, studies on HCC, CRC, and PC administered multi-kinase inhibitors without genomic testing [89, 91–95]. Thus, it is challenging to identify the common FGFR alterations or the responder population characteristics. Responses to multi-kinase inhibitors result from the blockade of FGFR along with other receptors such as VEGFR and PDGFR. There is currently insufficient evidence to attribute the observed response or efficacy solely to a FGFR blockade. [92–95]. It is urgent to increase the quality of research by applying molecular diagnostic, especially when multi-kinase inhibitors are administered, to provide evidence about the efficacy in different patient populations. Based on our inclusion and exclusion criteria, only two studies were identified for each PC [94, 95] and CRC [92, 93]. These studies mainly involved smaller cohorts, except for the trial of Van Cutsem et al. [93]. Recruiting larger patient populations for future research is essential to improve accuracy and yield statistically significant results [86, 92]. Due to the low number of studies investigating the FGFR-targeted therapy in PC and CRC, there is weak evidential base for FGFR as a prognostic marker for these cancers. Further clinical trials administering FGFR inhibitors for CRC and PC are recommended.

Molecular diagnosis was a strength of many studies, while the absence of it was a limitation in other trials. The most used genomic testing methods in the reviewed studies were FISH [80, 82, 83, 85] and NGS [79, 85–87]. However, inconsistencies between FISH, RT-qPCR, and IHC results emphasizes the need for a more reliable gold standard molecular test to detect FGFR alterations [85, 90]. Future clinical trials should implement a more consistent and standardized molecular testing for FGFR status. Investigating fusion partners, as conducted in multiple trials, can deepen the knowledge about genomic profiles [79–82] and enhance the specificity of targeted therapy. Genomic testing of the responder and non-responder population is crucial to make therapeutic recommendations. Few studies evaluated responses across different types of FGFR alteration [79, 83, 88]. The FIGHT-202 study provided the evidence applying the intervention to all cohorts [79]. As a result, clinicians can make recommendations and identify which patients benefit from the FGFR-targeted therapy. These finding support clinicians in making informed recommendations and identify the responder population [27, 79]. It is important to acknowledge that some patients may respond to targeted therapy due to unknown reasons making it difficult to develop definitive guidelines. Nevertheless, guidelines provide a framework for broader patient populations.

Most reviewed studies administered FGFR-targeted therapy as second or third line of treatment [79–82, 92]. Conducting trials with treatment naive patients may guide therapeutic guidelines. The absence of comparator groups in specific studies complicated the evaluation of whether the efficacy of the FGFR-targeted therapy outweighs the benefits of conventional therapy. Future research could compare targeted therapy to chemotherapy. Combination therapies such as trials combining FGFR-targeted therapy with chemotherapy [88, 92, 95] or with PD-1 inhibitors [86] can further investigate whether they offer improved outcomes compared to the FGFR-targeted therapy as monotherapy. For example, Ettrich et al. noted trends of improvement but failed to achieve statistically significant results [92]. Geographical differences are underscored by Shi et al. replicating the FIGHT-202 study. This study was conducted in China, and the outcomes slightly differ in the genomic of fusion partners, PFS and OS [79, 82]. International and multicentral trials with larger and more ethnically diverse cohorts reflect the global population more accurately.

While this systematic review comprehensively analysed existing clinical trials evaluating the predictive role of FGFR in gastrointestinal malignancies, ongoing trials may contribute to new findings in the future. These studies could provide valuable insight into FGFR-targeted therapies. A summary of the current ongoing clinical trials in phase 2 and beyond, with known status, registered on ClinicalTrials.gov is provided below. The search was conducted on 6 March 2025 on ClinicalTrials.gov using the term “FGFR.” All trials investigating various types of solid tumours were manually excluded to specifically assess the research focus on the GI cancers this review evaluated. The trend in ongoing clinical trials predominantly illustrates single-centre studies. Currently, participant numbers remain unknown, preventing any assessment of an increasing or decreasing pattern. Additionally, more combination therapies are being implemented. As demonstrated in this review, there is one ongoing clinical trial ongoing for both PC and CRC, while research in FGFR-targeted therapies is evolving for CC, GC/OC, and HCC. This further reinforces the recommendations outlined in the review.

Regarding the trials completed since the search process was terminated for this systematic review in 2023, only one trial in phase 2 and beyond was available as full-text. This study evaluated the efficacy and safety profile of futibatinib in patients with gastric or gastroesophageal junction cancer carrying FGFR2 amplifications [97]. However, the effect of futibanitib was not assessed in GC/OC in any ongoing clinical trial or the studies reviewed in this paper. The limited availability of published clinical trials highlights the need for further research on FGFR-targeted therapies in GI malignancies (Table 5).

**Table 5:** Registered ongoing clinical trials with FGFR-targeted therapy in GI malignancies on ClinicalTrials.gov

Study title	NCT number and phase	Status	GI cancers	Intervention	Location
*A single-arm phase II exploratory clinical study of pemigatinib in the treatment of advanced gastrointestinal cancer (excluding biliary tract cancer) patients with FGFR alterations who have failed standard therapy*[98]	NCT05559775 phase 2	Recruiting	GI cancers (exclusion CC)	Pemigatinib	China
*Pemigatinib in the advanced gastrointestinal cancer with FGFR 1–3 alterations*[99]	NCT05651672 phase 2	Recruiting	GI cancers	Pemigatinib	China
*Regorafenib combined with PD-1 inhibitor therapy for second-line treatment of hepatocellular carcinoma*[100]	NCT05048017 phase 2	Recruiting	HCC	Regorafenib PD-1 Inhibitor	China
*Futibatinib and pembrolizumab for treatment of advanced or metastatic FGF19 positive BCLC stage A, B, or C liver cancer*[101]	NCT04828486 phase 2	Active, not recruiting	HCC	Futibatinib Pembrolizumab Quality-of-life assessment	USA
*Lenvatinib combined toripalimab in advanced hepatocellular carcinoma*[102]	NCT04368078 phase 2	Recruiting	HCC	Toripalimab + lenvatinib	China
*A phaseI/II study of simmitinib or irinotecan liposomes combined with DP303c in gastric adenocarcinoma or gastroesophageal junction adenocarcinoma*[103]	NCT06577376 phase1/2	Not yet recruiting	GC/OC (sdenocarcinoma)	DP303c Simmitinib Irinotecan Paclitaxel ordocetaxel	N/A
*A study of simmitinib versus chemotherapy for participants with advanced oesophageal squamous cell carcinoma*[104]	NCT06656091 phase 3	Not yet recruiting	OC (squamous cell)	Simmitinib docetaxel or irinotecan	N/A
*Cadonilimab combined with anlotinib followed by radiotherapy in recurrent or metastatic esophageal squamous cell carcinoma (CAR-RMEC)*[105]	NCT06681285 phase 2	Not yet recruiting	OC (squamous cell)	Cadonilimab Anlotinib	China
Bemarituzumab or placebo plus chemotherapy in gastric cancers with fibroblast growth factor receptor 2b (FGFR2b) overexpression (FORTITUDE-101)[106]	NCT05052801 phase 3	Active, not recruiting	GC	Bemarituzumab mFOLFOX6 Placebo	Multicentre: USA, South Africa, Australia, Belgium, Brazil, China, Bulgaria, Canada, Chile, Denmark, Colombia, Czechia, Estonia, Spain, France, Greece, Hungary, Ireland, Israel, Italy, Japan, Republic of Korea, Lithuania, Malaysia, Peru, Taiwan, Mexico, Norway, Sweden, Portugal, Poland, Romania, Turkey, Singapore, Thailand, Argentina
*A single arm, phase II exploratory clinical study of pemitinib in advanced gastric cancer with previous standard therapy failure the FGFR variant*[107]	NCT05997459 phase 2	Not yet recruiting	GC	Pemigatinib	China
*Perioperative surufatinib plus sintilimab combined with chemotherapy in gastric/gastroesophageal junction adenocarcinoma*[108]	NCT06447636 phase 2	Not yet recruiting	GC/OC	Surufatinib Sintilimab Oxaliplatin	China
*Pemigatinib for the treatment of metastatic or unresectable colorectal cancer harboring FGFR alterations*[109]	NCT04096417 phase 2	Active, not recruiting	CRC	Pemigatinib Quality-of-life assessment	Multicentre: USA
*Surufatinib combined with KN046 and AG regimen chemotherapy as first-line treatment for unresectable advanced pancreatic cancer*[110]	NCT05832892 phase1/2	Recruiting	PC	Surufatinib KN046 Gemcitabine Paclitaxel	China
*Pembrolizumab in combination with lenvatinib in patients with advanced biliary tract carcinoma*[111]	NCT04550624 phase 2	Recruiting	CC	Pembrolizumab Lenvatinib	N/A
*Study of futibatinib in patients with advanced cholangiocarcinoma with FGFR2 fusion or rearrangement (FOENIX-CCA4)*[112]	NCT05727176 phase 2	Recruiting	CC	Futibatinib	Multicentre: USA, Spain. Portugal, Poland, Republic of Korea, Japan, Italy, Hong Kong, China, Brazil, Australia, Argentina
*Phase 2 study of HMPL-453 tartrate in advanced intrahepatic cholangiocarcinoma*[113]	NCT04353375 phase 2	Recruiting	CC	HMPL-453	China
*A study of E7090 in participants with unresectable advanced or metastatic cholangiocarcinoma with fibroblast growth factor receptor (FGFR) 2 gene fusion*[114]	NCT04238715 phase 2	Active, not recruiting	CC	E7090	Multicentre: China and Japan
*Study of TT-00420 (tinengotinib) in subjects with cholangiocarcinoma who failed or relapsed to chemotherapy and FGFR inhibitor*[115]	NCT06057571 phase 2	Recruiting	CC	Tinengotinib	Multicentre: China
*Pemigatinib combined with durvalumab for previously treated biliary tract carcinoma*[116]	NCT06530823 phase 2	Not yet recruiting	CC	Pemigatinib + durvalumab	N/A
*Study of tinengotinib vs. physician’s choice a treatment of subjects with FGFR-altered in cholangiocarcinoma (FIRST-308)*[117]	NCT05948475 phase 3	Recruiting	CC	Tinengotinib + physician’s choice	Multicentre: USA, Austria, Poland, Belgium, Netherlands, Portugal, UK, Taiwan. Spain, Italy, Republic of Korea, Germany, France
*A study to evaluate the efficacy and safety of pemigatinib versus chemotherapy in unresectable or metastatic cholangiocarcinoma (FIGHT-302)*[118]	NCT03656536 phase 3	Active, not recruiting	CC	Pemigatinib Gemcitabine Cisplatin	Multicentre: USA, Austria, China, Belgium, Canada, Denmark, Israel, Finland, Ireland, Netherlands, Poland, Portugal, UK, Spain, Italy, Japan, Norway, Switzerland, Sweden, Germany, France

**Fig. 3:**
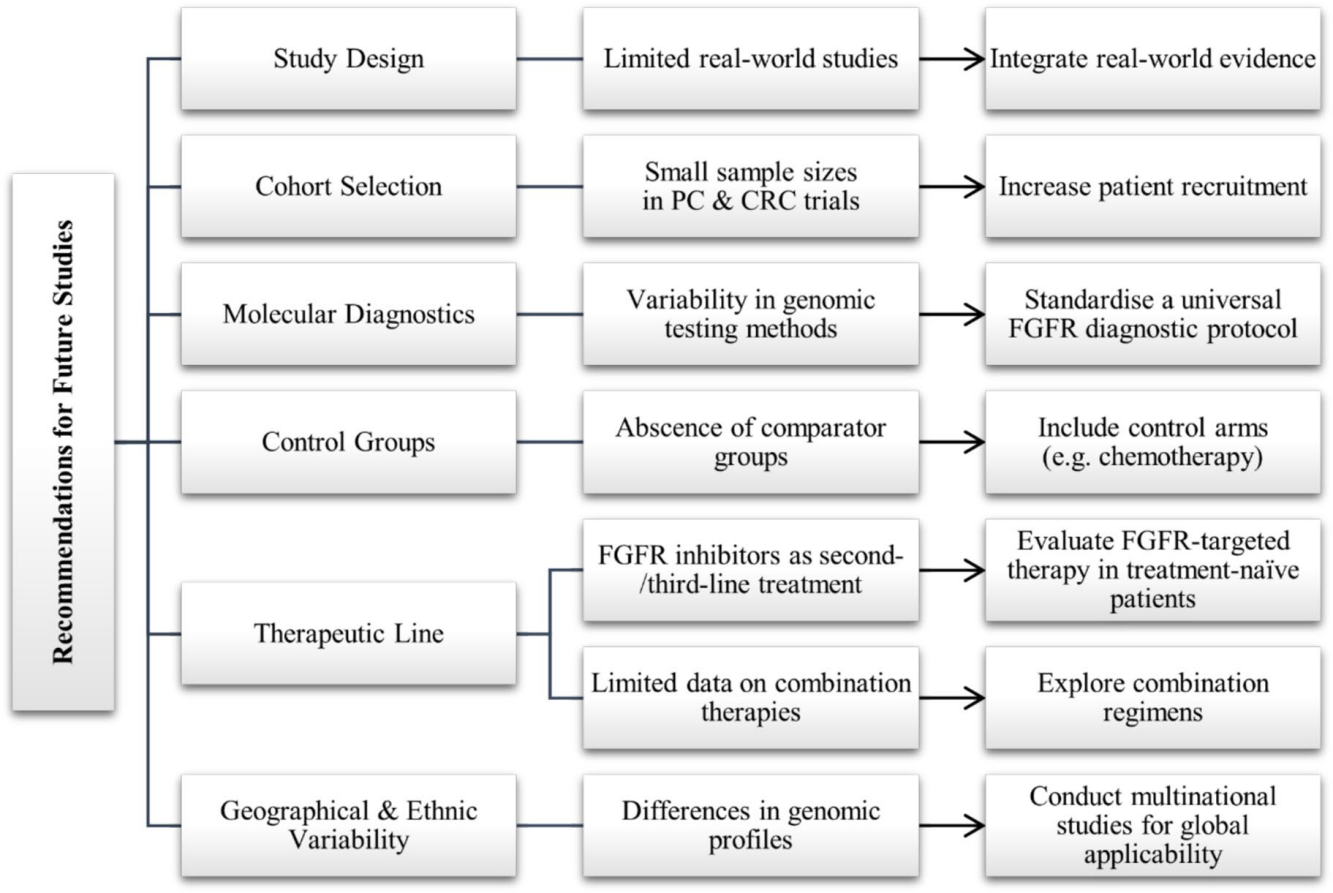
Methodological concerns and recommendations for FGFR-targeted therapy in GI malignancies

Figure 3 outlines key methodological challenges and recommendations in future research and trial design for FGFR-targeted therapy in gastrointestinal malignancies.

### Strength and Limitations

Quality assessment tools demonstrated a low risk of bias in all included studies. The time frame of the inclusion criterion resulted in the review focusing on relevant published data in the past 5 years. Limitations mainly concerned the study selection. As an inclusion criterion, only English and German publications were considered, leading to the exclusion of papers from other demographics. The unavailability of the full-text version of some of the trials also prevented the inclusion of many studies in the review. This review did not include real-world studies, suggesting that this analysis might not completely reflect the efficacy and establishment of FGFR-targeted therapies in routine clinical practice. The diversity of the FGFR inhibitors elicits vast options for treatment; yet, it makes the comparison of the published trials complex.

FGFR-targeted therapy is an underexplored field that requires greater research attention. Due to the limited number of systematic reviews on this topic, direct comparison with other analyses is challenging, which may be a limitation of this review. However, a literature review by Ratti et al. [119], published in 2023, primarily focused on toxicity, resistance mutations, and efficacy of FGFR-targeted drugs. Nevertheless, several key findings align with the themes discussed in this systematic review, particularly regarding the challenges with the standardization of genomic testing methods, lack of real-world data, and the need to explore combination therapies. There are minor differences between the analyses of Ratti et al. [119] and this review. While Ratti et al. [119] mentioned FGFR2 fusion as the most prevalent FGFR alteration in CC, they reported different predominant alterations for GC and HCC, namely FGFR4 mutations and FGFR3/4 amplifications, respectively. These findings were based on individual studies referenced. Like Ratti et al., this review considers FGFR-targeted therapies as an emerging intervention in precision medicine for GC and HCC.

## Conclusion

Overall, FGFR is a predictive marker for GI cancers. However, FGFR-targeted therapy has not been intensively established for all GI carcinomas as for CC. Phase 3 expansions are recommended for GC/OC and HCC as the efficacy of the FGFR-targeted therapy was demonstrated in patients with FGFR alterations. The limited number of studies on FGFR-targeted therapy for CRC and PC highlights the need for further research. Genomic testing identified specific FGFR alterations as more responsive and predictable for distinct GI malignancies. Future studies with larger cohorts and consistent molecular testing are essential to determine the predictive value of FGFR-targeted therapy for responder population.

## Supplementary Information

Below is the link to the electronic supplementary material.

ESM 1 (DOCX 50.1 KB)

## Data Availability

No datasets were generated or analysed during the current study.

## Abbreviations

GI: Gastrointestinal
CC: Cholangiocarcinoma
iCC: Intrahepatic cholangiocarcinoma
GC: Gastric cancer
OC: Oesophageal cancer
SCC: Squamous cell carcinoma
CRC: Colorectal cancer
PC: Pancreatic cancer
HCC: Hepatocellular carcinoma
FGFR: Fibroblast growth factor receptor
FGF: Fibroblast growth factor
NHS: National health service
CDC: The centers for disease control and prevention
NICE: The national institute for health and care excellence
EGFR: Epidermal growth factor receptor
HDI: Human development index
FDA: Food and drug administration
RTK: Receptor tyrosine kinase
CASP: Critical appraisal skills programme
MINORS: Methodological index for non-randomised studies
RCT: Randomised controlled trial
PRISMA: Preferred reporting items for systematic reviews and meta-analyses
OS: Overall survival
PFS: Progression-free survival
ORR: Objective response rate
CR: Complete response/responses
PR: Partial response/responses
NGS: Next generation sequencing
ctDNA: Circulating tumor DNA
pts: Patients
M: Median
FISH: Fluorescence in situ hybridisation
RT-qPCR: Quantitative reverse transcription polymerase chain reaction
BICR: Blinded independent central review
BICC1: Bicaudal-C1
I: Investigator
IRRC: Independent radiology review committee
B: Bemarituzumab
P: Placebo
MSK-IMPACT: Memorial sloan kettering-integrated mutation profiling of actionable cancer targets
IHC: Immunohistochemistry
PD-1: Programmed cell death protein 1
FGFR-M: FGFR mutation
FGFR-W: FGFR wild-type
KLB: β‐Klotho
mRECIST: Modified response evaluation criteria in solid tumours
RECIST v.1.1: Response evaluation criteria in solid tumours version 1.1
L: Lenvatinib
L + N: Lenvatinib + nivolumab
S: Surufatinib
mFOLFOX6: 5-Fluorouracil, leucovorin, and oxaliplatin
ELISA: Enzyme-linked immunoassay
FGF23: Fibroblast growth factor-23
bFGF: Basic fibroblast growth factor
NET: Neuroendocrine tumours
VEGFR: Vascular endothelial growth factor receptor
CSF-1R: Colony-stimulating factor-1 receptor
CNT: Cannot tell
