# Supplementary material for: FGFR as a Predictive Marker for Targeted Therapy in Gastrointestinal Malignancies: A Systematic Review
Source: J Gastrointest Cancer. 2025 Apr 9;56(1):96. doi: 10.1007/s12029-025-01214-y (PMC11982104; doi:10.1007/s12029-025-01214-y)
Supplement: Supplementary file 1 — (DOCX 50.1 KB) [file 12029_2025_1214_MOESM1_ESM.docx]

# **Appendices – Supplementary materials**

## **Appendix: Search Strategy**

**MEDLINE Ovid Search: ALL <1946 to September 24, 2023>**

| 1 | FGFR*.ti,ab. | 13046 |
| --- | --- | --- |
| 2 | "Fibroblast growth factor receptor".mp. [mp=title, book title, abstract, original title, name of substance word, subject heading word, floating sub-heading word, keyword heading word, organism supplementary concept word, protocol supplementary concept word, rare disease supplementary concept word, unique identifier, synonyms, population supplementary concept word, anatomy supplementary concept word] | 5898 |
| 3 | exp Receptors, Fibroblast Growth Factor/ | 11782 |
| 4 | receptor, fibroblast growth factor, type 1/ or receptor, fibroblast growth factor, type 2/ or receptor, fibroblast growth factor, type 3/ or receptor, fibroblast growth factor, type 4/ | 6702 |
| 5 | 1 or 2 or 3 or 4 | 18550 |
| 6 | ((predictive or prognostic) adj2 (marker* or factor*)).mp. | 210504 |
| 7 | biomarker*.mp. [mp=title, book title, abstract, original title, name of substance word, subject heading word, floating sub-heading word, keyword heading word, organism supplementary concept word, protocol supplementary concept word, rare disease supplementary concept word, unique identifier, synonyms, population supplementary concept word, anatomy supplementary concept word] | 779986 |
| 8 | exp Biomarkers/ | 885931 |
| 9 | exp Prognosis/ | 1930208 |
| 10 | 6 or 7 or 8 or 9 | 2935734 |
| 11 | "line-directed therap*".mp. [mp=title, book title, abstract, original title, name of substance word, subject heading word, floating sub-heading word, keyword heading word, organism supplementary concept word, protocol supplementary concept word, rare disease supplementary concept word, unique identifier, synonyms, population supplementary concept word, anatomy supplementary concept word] | 0 |
| 12 | "targeted therap*".mp. [mp=title, book title, abstract, original title, name of substance word, subject heading word, floating sub-heading word, keyword heading word, organism supplementary concept word, protocol supplementary concept word, rare disease supplementary concept word, unique identifier, synonyms, population supplementary concept word, anatomy supplementary concept word] | 100790 |
| 13 | therap*.mp. [mp=title, book title, abstract, original title, name of substance word, subject heading word, floating sub-heading word, keyword heading word, organism supplementary concept word, protocol supplementary concept word, rare disease supplementary concept word, unique identifier, synonyms, population supplementary concept word, anatomy supplementary concept word] | 7282621 |
| 14 | treatment*.mp. [mp=title, book title, abstract, original title, name of substance word, subject heading word, floating sub-heading word, keyword heading word, organism supplementary concept word, protocol supplementary concept word, rare disease supplementary concept word, unique identifier, synonyms, population supplementary concept word, anatomy supplementary concept word] | 6174486 |
| 15 | exp Diagnosis/ | 9403505 |
| 16 | exp Therapeutics/ | 5249723 |
| 17 | drug therapy/ or antineoplastic protocols/ or chemoprevention/ or chemoradiotherapy/ or chemotherapy, adjuvant/ or consolidation chemotherapy/ or molecular targeted therapy/ | 134304 |
| 18 | 11 or 12 or 13 or 14 or 15 or 16 or 17 | 17413092 |
| 19 | "malignant tumo?r*".mp. [mp=title, book title, abstract, original title, name of substance word, subject heading word, floating sub-heading word, keyword heading word, organism supplementary concept word, protocol supplementary concept word, rare disease supplementary concept word, unique identifier, synonyms, population supplementary concept word, anatomy supplementary concept word] | 68996 |
| 20 | cancer*.mp. [mp=title, book title, abstract, original title, name of substance word, subject heading word, floating sub-heading word, keyword heading word, organism supplementary concept word, protocol supplementary concept word, rare disease supplementary concept word, unique identifier, synonyms, population supplementary concept word, anatomy supplementary concept word] | 2309624 |
| 21 | Malignan*.mp. [mp=title, book title, abstract, original title, name of substance word, subject heading word, floating sub-heading word, keyword heading word, organism supplementary concept word, protocol supplementary concept word, rare disease supplementary concept word, unique identifier, synonyms, population supplementary concept word, anatomy supplementary concept word] | 702523 |
| 22 | Tumo?r*.mp. [mp=title, book title, abstract, original title, name of substance word, subject heading word, floating sub-heading word, keyword heading word, organism supplementary concept word, protocol supplementary concept word, rare disease supplementary concept word, unique identifier, synonyms, population supplementary concept word, anatomy supplementary concept word] | 2533139 |
| 23 | Carcinoma*.mp. [mp=title, book title, abstract, original title, name of substance word, subject heading word, floating sub-heading word, keyword heading word, organism supplementary concept word, protocol supplementary concept word, rare disease supplementary concept word, unique identifier, synonyms, population supplementary concept word, anatomy supplementary concept word] | 1021541 |
| 24 | Neoplas*.mp. [mp=title, book title, abstract, original title, name of substance word, subject heading word, floating sub-heading word, keyword heading word, organism supplementary concept word, protocol supplementary concept word, rare disease supplementary concept word, unique identifier, synonyms, population supplementary concept word, anatomy supplementary concept word] | 3393675 |
| 25 | exp Neoplasms/ | 3878513 |
| 26 | exp Cell Line, Tumor/ | 585050 |
| 27 | 19 or 20 or 21 or 22 or 23 or 24 or 25 or 26 | 5440992 |
| 28 | ((gastrointestinal or gastric or colorectal or biliary or cholangio or liver or hepatic or pancreatic) adj3 (cancer* or tumo?r* or neoplasm* or carcinoma*)).mp. [mp=title, book title, abstract, original title, name of substance word, subject heading word, floating sub-heading word, keyword heading word, organism supplementary concept word, protocol supplementary concept word, rare disease supplementary concept word, unique identifier, synonyms, population supplementary concept word, anatomy supplementary concept word] | 619780 |
| 29 | ("gastric cancer" or "pancreatic cancer" or "liver cancer" or "biliary tract cancer" or "cholangiocarcinoma" or "colorectal cancer").mp. [mp=title, book title, abstract, original title, name of substance word, subject heading word, floating sub-heading word, keyword heading word, organism supplementary concept word, protocol supplementary concept word, rare disease supplementary concept word, unique identifier, synonyms, population supplementary concept word, anatomy supplementary concept word] | 307398 |
| 30 | (stomach or colorectal or colon or "biliary tract" or liver or pancreas).mp. [mp=title, book title, abstract, original title, name of substance word, subject heading word, floating sub-heading word, keyword heading word, organism supplementary concept word, protocol supplementary concept word, rare disease supplementary concept word, unique identifier, synonyms, population supplementary concept word, anatomy supplementary concept word] | 1971289 |
| 31 | exp Digestive System/ | 1306059 |
| 32 | digestive system neoplasms/ or exp digestive system diseases/ | 1966451 |
| 33 | 28 or 29 or 30 or 31 or 32 | 3371171 |
| 34 | 5 and 10 and 18 and 27 and 33 | 586 |
| 35 | 34 not (exp animal/ not exp human/) | 574 |
| 36 | Randomized controlled trials as Topic/ | 164134 |
| 37 | Randomized controlled trial/ | 600435 |
| 38 | Random allocation/ | 106965 |
| 39 | Double blind method/ | 176190 |
| 40 | Single blind method/ | 32936 |
| 41 | Clinical trial/ | 538862 |
| 42 | exp Clinical Trials as Topic/ | 384743 |
| 43 | or/36-42 | 1327816 |
| 44 | (clinic$ adj trial$1).tw. | 485912 |
| 45 | ((singl$ or doubl$ or treb$ or tripl$) adj (blind$3 or mask$3)).tw. | 199507 |
| 46 | Placebos/ | 35932 |
| 47 | Placebo$.tw. | 249429 |
| 48 | Randomly allocated.tw. | 36933 |
| 49 | (allocated adj2 random).tw. | 839 |
| 50 | or/44-49 | 790779 |
| 51 | 43 or 50 | 1693649 |
| 52 | Case report.tw. | 404449 |
| 53 | Letter/ | 1229808 |
| 54 | Historical article/ | 369445 |
| 55 | Review of reported cases.pt. | 0 |
| 56 | Review, multicase.pt. | 0 |
| 57 | or/52-56 | 1984441 |
| 58 | 51 not 57 | 1654300 |
| 59 | 35 and 58 | 107 |
| 60 | limit 59 to (yr="2018 -Current" and (english or german)) | 61 |

MEDLINE: not (exp animals/ not humans.sh.) adapted from UCLA library and other sources

**Embase Classic+Embase Search: <1947 to 2023 September 23>**

| 1 | FGFR*.ti,ab. | 21893 |
| --- | --- | --- |
| 2 | "Fibroblast growth factor receptor".mp. [mp=title, abstract, heading word, drug trade name, original title, device manufacturer, drug manufacturer, device trade name, keyword heading word, floating subheading word, candidate term word] | 28586 |
| 3 | exp Receptors, Fibroblast Growth Factor/ | 9147 |
| 4 | receptor, fibroblast growth factor, type 1/ or receptor, fibroblast growth factor, type 2/ or receptor, fibroblast growth factor, type 3/ or receptor, fibroblast growth factor, type 4/ | 18799 |
| 5 | 1 or 2 or 3 or 4 | 33771 |
| 6 | ((predictive or prognostic) adj2 (marker* or factor*)).mp. | 325471 |
| 7 | biomarker*.mp. [mp=title, abstract, heading word, drug trade name, original title, device manufacturer, drug manufacturer, device trade name, keyword heading word, floating subheading word, candidate term word] | 630577 |
| 8 | exp Biomarkers/ | 436135 |
| 9 | exp Prognosis/ | 965220 |
| 10 | 6 or 7 or 8 or 9 | 1755429 |
| 11 | "line-directed therap*".mp. [mp=title, abstract, heading word, drug trade name, original title, device manufacturer, drug manufacturer, device trade name, keyword heading word, floating subheading word, candidate term word] | 1 |
| 12 | "targeted therap*".mp. [mp=title, abstract, heading word, drug trade name, original title, device manufacturer, drug manufacturer, device trade name, keyword heading word, floating subheading word, candidate term word] | 142604 |
| 13 | therap*.mp. [mp=title, abstract, heading word, drug trade name, original title, device manufacturer, drug manufacturer, device trade name, keyword heading word, floating subheading word, candidate term word] | 10953182 |
| 14 | treatment*.mp. [mp=title, abstract, heading word, drug trade name, original title, device manufacturer, drug manufacturer, device trade name, keyword heading word, floating subheading word, candidate term word] | 9192274 |
| 15 | exp Diagnosis/ | 8585873 |
| 16 | exp Therapeutics/ | 10865472 |
| 17 | drug therapy/ or antineoplastic protocols/ or chemoprevention/ or chemoradiotherapy/ or chemotherapy, adjuvant/ or consolidation chemotherapy/ or molecular targeted therapy/ | 1144450 |
| 18 | 11 or 12 or 13 or 14 or 15 or 16 or 17 | 21982881 |
| 19 | "malignant tumo?r*".mp. [mp=title, abstract, heading word, drug trade name, original title, device manufacturer, drug manufacturer, device trade name, keyword heading word, floating subheading word, candidate term word] | 100071 |
| 20 | cancer*.mp. [mp=title, abstract, heading word, drug trade name, original title, device manufacturer, drug manufacturer, device trade name, keyword heading word, floating subheading word, candidate term word] | 4673472 |
| 21 | Malignan*.mp. [mp=title, abstract, heading word, drug trade name, original title, device manufacturer, drug manufacturer, device trade name, keyword heading word, floating subheading word, candidate term word] | 1199672 |
| 22 | Tumo?r*.mp. [mp=title, abstract, heading word, drug trade name, original title, device manufacturer, drug manufacturer, device trade name, keyword heading word, floating subheading word, candidate term word] | 4112896 |
| 23 | Carcinoma*.mp. [mp=title, abstract, heading word, drug trade name, original title, device manufacturer, drug manufacturer, device trade name, keyword heading word, floating subheading word, candidate term word] | 1532993 |
| 24 | Neoplas*.mp. [mp=title, abstract, heading word, drug trade name, original title, device manufacturer, drug manufacturer, device trade name, keyword heading word, floating subheading word, candidate term word] | 1252057 |
| 25 | exp Neoplasms/ | 6000706 |
| 26 | exp Cell Line, Tumor/ | 477443 |
| 27 | 19 or 20 or 21 or 22 or 23 or 24 or 25 or 26 | 7820179 |
| 28 | ((gastrointestinal or gastric or colorectal or biliary or cholangio or liver or hepatic or pancreatic) adj3 (cancer* or tumo?r* or neoplasm* or carcinoma*)).mp. [mp=title, abstract, heading word, drug trade name, original title, device manufacturer, drug manufacturer, device trade name, keyword heading word, floating subheading word, candidate term word] | 909082 |
| 29 | ("gastric cancer" or "pancreatic cancer" or "liver cancer" or "biliary tract cancer" or "cholangiocarcinoma" or "colorectal cancer").mp. [mp=title, abstract, heading word, drug trade name, original title, device manufacturer, drug manufacturer, device trade name, keyword heading word, floating subheading word, candidate term word] | 537815 |
| 30 | (stomach or colorectal or colon or "biliary tract" or liver or pancreas).mp. [mp=title, abstract, heading word, drug trade name, original title, device manufacturer, drug manufacturer, device trade name, keyword heading word, floating subheading word, candidate term word] | 3516358 |
| 31 | exp Digestive System/ | 1871606 |
| 32 | digestive system neoplasms/ or exp digestive system diseases/ | 4092544 |
| 33 | 28 or 29 or 30 or 31 or 32 | 5716968 |
| 34 | 5 and 10 and 18 and 27 and 33 | 1619 |
| 35 | 34 not ((exp animal/ or animal experiment/ or nonhuman/) not (exp human/ or human experiment/)) | 1584 |
| 36 | Clinical trial/ | 1091004 |
| 37 | Randomized controlled trial/ | 786260 |
| 38 | Randomization/ | 98682 |
| 39 | Single blind procedure/ | 51835 |
| 40 | Double blind procedure/ | 213353 |
| 41 | Crossover procedure/ | 75752 |
| 42 | Placebo/ | 413202 |
| 43 | Randomi?ed controlled trial$.tw. | 326138 |
| 44 | Rct.tw. | 54309 |
| 45 | Random allocation.tw. | 2644 |
| 46 | Randomly allocated.tw. | 45748 |
| 47 | Allocated randomly.tw. | 2977 |
| 48 | (allocated adj2 random).tw. | 1036 |
| 49 | Single blind$.tw. | 31889 |
| 50 | Double blind$.tw. | 250426 |
| 51 | ((treble or triple) adj blind$).tw. | 1963 |
| 52 | Placebo$.tw. | 373072 |
| 53 | Prospective study/ | 882439 |
| 54 | or/36-53 | 2805737 |
| 55 | Case study/ | 105631 |
| 56 | Case report.tw. | 567193 |
| 57 | Abstract report/ or letter/ | 1300938 |
| 58 | or/55-57 | 1958640 |
| 59 | 54 not 58 | 2739671 |
| 60 | 35 and 59 | 259 |
| 61 | limit 60 to ((english or german) and yr="2018 -Current") | 140 |

Embase: not ((exp animal/ or animal experiment/ or nonhuman/) not (exp human/ or human experiment/)) [Adapted from https://www.cochranelibrary.com/central/central-creation]

**PubMed search: 22nd of Sep 2023**

(FGFR* OR "fibroblast growth factor receptor*" [Title/Abstract]) AND (cancer* OR malignan* OR neoplas* OR tumor* OR tumour* OR carcinoma*) AND (gastrointestinal OR gastric OR colorectal OR biliary OR cholangio OR liver OR pancreatic OR hepatic OR "gastrointestinal system*" OR "digestive system*")

**CINAHL:** Sunday, September 24, 2023 1:31:22 PM

| **#** | **Query** | **Limiters/Expanders** | **Last Run Via** | **Results** |
| --- | --- | --- | --- | --- |
| S36 | S34 NOT S35 | Limiters - Published Date: 20180101-20231231 Expanders - Apply equivalent subjects Search modes - Find all my search terms | Interface - EBSCOhost Research Databases Search Screen - Advanced Search Database - CINAHL Plus with Full Text | 66 |
| S35 | (((MH "Animals+") OR (MH "Animal Studies") OR (TI "animal model*")) NOT (MH "human")) | Expanders - Apply equivalent subjects Search modes - Find all my search terms | Interface - EBSCOhost Research Databases Search Screen - Advanced Search Database - CINAHL Plus with Full Text | 216,072 |
| S34 | S5 AND S10 AND S18 AND S27 AND S33 | Expanders - Apply equivalent subjects Search modes - Find all my search terms | Interface - EBSCOhost Research Databases Search Screen - Advanced Search Database - CINAHL Plus with Full Text | 95 |
| S33 | S28 OR S29 OR S30 OR S31 OR S32 | Expanders - Apply equivalent subjects Search modes - Find all my search terms | Interface - EBSCOhost Research Databases Search Screen - Advanced Search Database - CINAHL Plus with Full Text | 434,980 |
| S32 | (MH "digestive system neoplasms") OR (MH "digestive system diseases+") | Expanders - Apply equivalent subjects Search modes - Find all my search terms | Interface - EBSCOhost Research Databases Search Screen - Advanced Search Database - CINAHL Plus with Full Text | 310,667 |
| S31 | (MH "Digestive System+") | Expanders - Apply equivalent subjects Search modes - Find all my search terms | Interface - EBSCOhost Research Databases Search Screen - Advanced Search Database - CINAHL Plus with Full Text | 94,349 |
| S30 | (stomach OR colorectal OR colon OR "biliary tract" OR liver OR pancreas) | Expanders - Apply equivalent subjects Search modes - Find all my search terms | Interface - EBSCOhost Research Databases Search Screen - Advanced Search Database - CINAHL Plus with Full Text | 216,676 |
| S29 | ("gastric cancer" or "pancreatic cancer" or "liver cancer" or "biliary tract cancer" or "cholangiocarcinoma" or "colorectal cancer") | Expanders - Apply equivalent subjects Search modes - Find all my search terms | Interface - EBSCOhost Research Databases Search Screen - Advanced Search Database - CINAHL Plus with Full Text | 87,159 |
| S28 | ((gastrointestinal OR gastric OR colorectal OR cholangio) N3 (cancer* OR tumo#r* OR neoplasm* OR carcinoma*)) | Expanders - Apply equivalent subjects Search modes - Find all my search terms | Interface - EBSCOhost Research Databases Search Screen - Advanced Search Database - CINAHL Plus with Full Text | 62,086 |
| S27 | S19 OR S20 OR S21 OR S22 OR S23 OR S24 OR S25 OR S26 | Expanders - Apply equivalent subjects Search modes - Find all my search terms | Interface - EBSCOhost Research Databases Search Screen - Advanced Search Database - CINAHL Plus with Full Text | 929,011 |
| S26 | (MH "Cell Line, Tumor+") | Expanders - Apply equivalent subjects Search modes - Find all my search terms | Interface - EBSCOhost Research Databases Search Screen - Advanced Search Database - CINAHL Plus with Full Text | 21,022 |
| S25 | (MH Neoplasms+) | Expanders - Apply equivalent subjects Search modes - Find all my search terms | Interface - EBSCOhost Research Databases Search Screen - Advanced Search Database - CINAHL Plus with Full Text | 661,193 |
| S24 | Neoplas* | Expanders - Apply equivalent subjects Search modes - Find all my search terms | Interface - EBSCOhost Research Databases Search Screen - Advanced Search Database - CINAHL Plus with Full Text | 569,034 |
| S23 | Carcinoma* | Expanders - Apply equivalent subjects Search modes - Find all my search terms | Interface - EBSCOhost Research Databases Search Screen - Advanced Search Database - CINAHL Plus with Full Text | 135,248 |
| S22 | Tumo#r* | Expanders - Apply equivalent subjects Search modes - Find all my search terms | Interface - EBSCOhost Research Databases Search Screen - Advanced Search Database - CINAHL Plus with Full Text | 320,441 |
| S21 | Malignan* | Expanders - Apply equivalent subjects Search modes - Find all my search terms | Interface - EBSCOhost Research Databases Search Screen - Advanced Search Database - CINAHL Plus with Full Text | 94,531 |
| S20 | cancer* | Expanders - Apply equivalent subjects Search modes - Find all my search terms | Interface - EBSCOhost Research Databases Search Screen - Advanced Search Database - CINAHL Plus with Full Text | 555,663 |
| S19 | "malignant tumo#r*" | Expanders - Apply equivalent subjects Search modes - Find all my search terms | Interface - EBSCOhost Research Databases Search Screen - Advanced Search Database - CINAHL Plus with Full Text | 6,354 |
| S18 | S11 OR S12 OR S13 OR S14 OR S15 OR S16 OR S17 | Expanders - Apply equivalent subjects Search modes - Find all my search terms | Interface - EBSCOhost Research Databases Search Screen - Advanced Search Database - CINAHL Plus with Full Text | 4,317,591 |
| S17 | (MH "drug therapy") OR (MH "antineoplastic protocols") OR (MH chemoprevention) OR (MH chemoradiotherapy) OR (MH "chemotherapy, adjuvant") OR (MH "consolidation chemotherapy") OR (MH "molecular targeted therapy") | Expanders - Apply equivalent subjects Search modes - Find all my search terms | Interface - EBSCOhost Research Databases Search Screen - Advanced Search Database - CINAHL Plus with Full Text | 34,143 |
| S16 | (MH Therapeutics+) | Expanders - Apply equivalent subjects Search modes - Find all my search terms | Interface - EBSCOhost Research Databases Search Screen - Advanced Search Database - CINAHL Plus with Full Text | 1,739,230 |
| S15 | (MH Diagnosis+) | Expanders - Apply equivalent subjects Search modes - Find all my search terms | Interface - EBSCOhost Research Databases Search Screen - Advanced Search Database - CINAHL Plus with Full Text | 2,189,064 |
| S14 | treatment* | Expanders - Apply equivalent subjects Search modes - Find all my search terms | Interface - EBSCOhost Research Databases Search Screen - Advanced Search Database - CINAHL Plus with Full Text | 1,366,196 |
| S13 | therap* | Expanders - Apply equivalent subjects Search modes - Find all my search terms | Interface - EBSCOhost Research Databases Search Screen - Advanced Search Database - CINAHL Plus with Full Text | 1,957,758 |
| S12 | "targeted therap*" | Expanders - Apply equivalent subjects Search modes - Find all my search terms | Interface - EBSCOhost Research Databases Search Screen - Advanced Search Database - CINAHL Plus with Full Text | 12,850 |
| S11 | "line-directed therap*" | Expanders - Apply equivalent subjects Search modes - Find all my search terms | Interface - EBSCOhost Research Databases Search Screen - Advanced Search Database - CINAHL Plus with Full Text | 2 |
| S10 | S6 OR S7 OR S8 OR S9 | Expanders - Apply equivalent subjects Search modes - Find all my search terms | Interface - EBSCOhost Research Databases Search Screen - Advanced Search Database - CINAHL Plus with Full Text | 652,970 |
| S9 | (MH Prognosis+) | Expanders - Apply equivalent subjects Search modes - Find all my search terms | Interface - EBSCOhost Research Databases Search Screen - Advanced Search Database - CINAHL Plus with Full Text | 555,226 |
| S8 | (MH Biomarkers+) | Expanders - Apply equivalent subjects Search modes - Find all my search terms | Interface - EBSCOhost Research Databases Search Screen - Advanced Search Database - CINAHL Plus with Full Text | 97 |
| S7 | biomarker* | Expanders - Apply equivalent subjects Search modes - Find all my search terms | Interface - EBSCOhost Research Databases Search Screen - Advanced Search Database - CINAHL Plus with Full Text | 88,447 |
| S6 | ((predictive OR prognostic) N2 (marker* OR factor*)) | Expanders - Apply equivalent subjects Search modes - Find all my search terms | Interface - EBSCOhost Research Databases Search Screen - Advanced Search Database - CINAHL Plus with Full Text | 41,602 |
| S5 | S1 OR S2 OR S3 OR S4 | Expanders - Apply equivalent subjects Search modes - Find all my search terms | Interface - EBSCOhost Research Databases Search Screen - Advanced Search Database - CINAHL Plus with Full Text | 1,579 |
| S4 | (MH "receptor, fibroblast growth factor, type 1") OR (MH "receptor, fibroblast growth factor, type 2") OR (MH "receptor, fibroblast growth factor, type 3") OR (MH "receptor, fibroblast growth factor, type 4") | Expanders - Apply equivalent subjects Search modes - Find all my search terms | Interface - EBSCOhost Research Databases Search Screen - Advanced Search Database - CINAHL Plus with Full Text | 6,037 |
| S3 | (MH "Receptors, Fibroblast Growth Factor+") | Expanders - Apply equivalent subjects Search modes - Find all my search terms | Interface - EBSCOhost Research Databases Search Screen - Advanced Search Database - CINAHL Plus with Full Text | 5,638 |
| S2 | "Fibroblast growth factor receptor*" | Expanders - Apply equivalent subjects Search modes - Find all my search terms | Interface - EBSCOhost Research Databases Search Screen - Advanced Search Database - CINAHL Plus with Full Text | 600 |
| S1 | (TI FGFR* OR AB FGFR*) | Expanders - Apply equivalent subjects Search modes - Find all my search terms | Interface - EBSCOhost Research Databases Search Screen - Advanced Search Database - CINAHL Plus with Full Text | 1,411 |

(((MH "Animals+") OR (MH "Animal Studies") OR (TI "animal model*")) NOT (MH "human")) adapted from Cochrane library

**Web of Science: 23^rd^ of Sep 2023**

((TI=FGFR* OR AB=FGFR*) OR TS="Fibroblast growth factor receptor" OR TS="Receptors, Fibroblast Growth Factor" OR TS="receptor, fibroblast growth factor, type 1" OR TS="receptor, fibroblast growth factor, type 2" OR TS="receptor, fibroblast growth factor, type 3" OR TS="receptor, fibroblast growth factor, type 4") AND (TS=((predictive OR prognostic ) "NEAR/2" (marker* OR factor* )) OR TS=biomarker* OR TS=Biomarkers OR TS=Prognosis) AND (TS="line-directed therap*" OR TS="targeted therap*" OR TS=therap* OR TS=treatment* OR TS=Diagnosis OR TS=Therapeutics OR TS="drug therapy" OR TS="antineoplastic protocols" OR TS=chemoprevention OR TS=chemoradiotherapy OR TS="chemotherapy, adjuvant" OR TS="consolidation chemotherapy" OR TS="molecular targeted therapy") AND (TS="malignant tumo$r*" OR TS=cancer* OR TS=Malignan* OR TS=Tumo$r* OR TS=Carcinoma* OR TS=Neoplas* OR TS=Neoplasms OR ALL="Cell Line, Tumor") AND (TS=((gastrointestinal OR gastric OR colorectal OR biliary or cholangio OR liver or hepatic OR pancreatic ) NEAR/3 (cancer* OR tumo$r* OR neoplasm* OR carcinoma* )) OR TS="Digestive System" OR TS="digestive system neoplasms" OR TS="digestive system diseases" OR TS="gastric cancer" OR TS="pancreatic cancer" OR TS="liver cancer" OR TS="biliary tract cancer" OR TS="cholangiocarcinoma" OR TS="colorectal cancer" OR TS=(stomach OR colorectal OR colon OR "biliary tract" OR liver OR pancreas)) NOT (ALL=animals NOT ALL=humans)

**Cochrane library: 24/09/2023 18:35:40**

| ID | Search | Hits |
| --- | --- | --- |
| #1 | (FGFR*):ti,ab,kw | 510 |
| #2 | "Fibroblast growth factor receptor":ti,ab,kw OR "Fibroblast growth factor receptors":ti,ab,kw | 242 |
| #3 | [mh "Receptors, Fibroblast Growth Factor"] | 87 |
| #4 | [mh ^"receptor, fibroblast growth factor, type 1"] OR [mh ^"receptor, fibroblast growth factor, type 2"] OR [mh ^"receptor, fibroblast growth factor, type 3"] OR [mh ^"receptor, fibroblast growth factor, type 4"] | 39 |
| #5 | #1 OR #2 OR #3 OR #4 | 621 |
| #6 | ((predictive:ti,ab,kw OR prognostic:ti,ab,kw) NEAR/2 (marker*:ti,ab,kw OR factor*:ti,ab,kw)) | 12142 |
| #7 | biomarker*:ti,ab,kw | 52279 |
| #8 | [mh Biomarkers] | 26929 |
| #9 | [mh Prognosis] | 203218 |
| #10 | #6 OR #7 OR #8 OR #9 | 255362 |
| #11 | ("line-directed" NEXT therap*):ti,ab,kw | 0 |
| #12 | ("targeted" NEXT therap*):ti,ab,kw | 3622 |
| #13 | therap*:ti,ab,kw | 912253 |
| #14 | treatment*:ti,ab,kw | 934327 |
| #15 | [mh Diagnosis] | 446376 |
| #16 | [mh Therapeutics] | 412617 |
| #17 | [mh ^"drug therapy"] OR [mh ^"antineoplastic protocols"] OR [mh ^chemoprevention] OR [mh ^chemoradiotherapy] OR [mh ^"chemotherapy, adjuvant"] OR [mh ^"consolidation chemotherapy"] OR [mh ^"molecular targeted therapy"] | 22727 |
| #18 | #11 OR #12 OR #13 OR #14 OR #15 OR #16 OR #17 | 1382167 |
| #19 | ("malignant" NEXT tumo?r*):ti,ab,kw | 2012 |
| #20 | cancer*:ti,ab,kw | 200104 |
| #21 | Malignan*:ti,ab,kw | 32454 |
| #22 | Tumo?r*:ti,ab,kw | 90550 |
| #23 | Carcinoma*:ti,ab,kw | 49397 |
| #24 | Neoplas*:ti,ab,kw | 110236 |
| #25 | [mh Neoplasms] | 112562 |
| #26 | [mh "Cell Line, Tumor"] | 591 |
| #27 | #19 OR #20 OR #21 OR #22 OR #23 OR #24 OR #25 OR #26 | 277201 |
| #28 | (gastrointestinal:ti,ab,kw OR gastric:ti,ab,kw OR colorectal:ti,ab,kw OR biliary:ti,ab,kw OR cholangio:ti,ab,kw OR liver:ti,ab,kw OR hepatic:ti,ab,kw pancreatic:ti,ab,kw) NEAR (cancer*:ti,ab,kw OR tumo?r*:ti,ab,kw OR neoplasm*:ti,ab,kw OR carcinoma*) | 43298 |
| #29 | "gastric cancer" OR "pancreatic cancer" OR "liver cancer" OR "biliary tract cancer" OR "cholangiocarcinoma" OR "colorectal cancer" | 29456 |
| #30 | (stomach:ti,ab,kw OR colorectal:ti,ab,kw OR colon:ti,ab,kw OR "biliary tract":ti,ab,kw OR liver:ti,ab,kw OR pancreas:ti,ab,kw) | 119850 |
| #31 | [mh "Digestive System"] | 23345 |
| #32 | [mh ^"digestive system neoplasms"] OR [mh "digestive system diseases"] | 75244 |
| #33 | #28 OR #29 OR #30 OR #31 OR #32 | 172987 |
| #34 | #5 AND #10 AND #18 AND #27 AND #33 with Publication Year from 2018 to 2023, in Trials | 32 |
| #35 | #34 NOT ([mh animals] NOT [mh humans]) | 32 |

## **Appendix: CASP RCT Checklist [78]**

| 1 | Did the study address a clearly focused research question? |
| --- | --- |
| 2 | Was the assignment of participants to interventions randomised? |
| 3 | Were all participants who entered the study accounted for at its conclusion? |
| 4 | Were the participants ‘blind’ to intervention they were given? |
| 5 | Were the investigators ‘blind’ to the intervention they were giving to participants? |
| 6 | Were the people assessing/analysing outcome/s ‘blinded’? |
| 7 | Were the study groups similar at the start of the randomized controlled trial? |
| 8 | Apart from the experimental intervention, did each study group receive the same level of care (that is, were they treated equally)? |
| 9 | Were the effects of intervention reported comprehensively? |
| 10 | Was the precision of the estimate of the intervention or treatment effect reported? |
| 11 | Do the benefits of the experimental intervention outweigh the harms and costs? |
| 12 | Can the results be applied to your local population/in your context? |
| 13 | Would the experimental intervention provide greater value to the people in your care than any of the existing interventions? |

**Table 1 Quality Assessment for included RCTs**

| **CASP Checklist for RCTs** | | | | | | | | | | | | | |
| --- | --- | --- | --- | --- | --- | --- | --- | --- | --- | --- | --- | --- | --- |
| **Articles** | 1 | 2 | 3 | 4 | 5 | 6 | 7 | 8 | 9 | 10 | 11 | 12 | 13 |
| Ettrich et al. [93] | Yes | Yes | No | Yes | Yes | Yes | Yes | Yes | Yes | Yes | CNT | Yes | CNT |
| Van Cutsem et al. [94] | Yes | Yes | No | Yes | Yes | Yes | Yes | Yes | Yes | Yes | CNT | Yes | CNT |
| Kudo et al. [90] | Yes | Yes | Yes | No | No | No | Yes | Yes | Yes | Yes | Yes | Yes | Yes |
| Yen et al. [92] | Yes | Yes | Yes | No | No | No | Yes | Yes | Yes | Yes | CNT | Yes | CNT |
| Wainberg et al. [89] | Yes | Yes | No | Yes | Yes | Yes | Yes | Yes | Yes | Yes | Yes | Yes | Yes |
| ***Green: Yes, Orange: Cannot tell (CNT), Red: No*** | | | | | | | | | | | | | |

## **Appendix: MINORS Checklist for non-RCTs [79]**

| 1 | Clearly stated aim(s) |
| --- | --- |
| 2 | Inclusion of consecutive patients including use of eligibility criteria |
| 3 | Prospective collection of data |
| 4 | Intervention standardised |
| 5 | Outcomes appropriate to the aim of the study |
| 6 | Unbiased assessment of study outcomes |
| 7 | Follow-up period appropriate to the aim of the study |
| 8 | Loss to follow up less than 5% |
| 9 | Prospective calculation of the study size |

**Table 2 Quality Assessment for included non-randomised clinical trials**

| **Modified MINORS criteria** | | | | | | | | | |
| --- | --- | --- | --- | --- | --- | --- | --- | --- | --- |
| **Articles** | 1 | 2 | 3 | 4 | 5 | 6 | 7 | 8 | 9 |
| Abou-Alfa et al. [80] | 2 | 2 | 2 | 2 | 2 | 2 | 2 | 2 | 2 |
| Javle et al. [81] | 2 | 2 | 2 | 2 | 2 | 2 | 2 | 2 | 2 |
| Goyal et al. [82] | 2 | 2 | 2 | 2 | 2 | 1 | 1 | 1 | 2 |
| Shi et al. [83] | 2 | 2 | 2 | 2 | 2 | 2 | 1 | 1 | 2 |
| Ahn et al. [84] | 2 | 2 | 2 | 2 | 2 | 2 | 1 | 1 | 2 |
| Won et al. [85] | 2 | 2 | 2 | 2 | 2 | 2 | 1 | 1 | 0 |
| Tsimafeyeu et al. [86] | 2 | 2 | 2 | 2 | 2 | 2 | 2 | 2 | 2 |
| Jiang et al. [87] | 2 | 2 | 2 | 2 | 2 | 2 | 1 | 1 | 2 |
| Catenacci et al. [88] | 2 | 2 | 2 | 2 | 2 | 2 | 2 | 2 | 2 |
| Kim et al. [28] | 2 | 2 | 2 | 2 | 2 | 2 | 2 | 2 | 0 |
| Chan et al. [91] | 2 | 2 | 2 | 2 | 2 | 2 | 2 | 2 | 2 |
| Xu et al. [95] | 2 | 2 | 2 | 2 | 2 | 2 | 1 | 2 | 2 |
| Ma et al. [96] | 2 | 2 | 2 | 2 | 2 | 2 | 1 | 1 | 0 |
| ***Green: Yes score of 2, Orange: Unclear score of 1, Red: No score of 0*** | | | | | | | | | |
